# Substitutions in the Amino-Terminal Tail of Neurospora Histone H3 Have Varied Effects on DNA Methylation

**DOI:** 10.1371/journal.pgen.1002423

**Published:** 2011-12-29

**Authors:** Keyur K. Adhvaryu, Emanuela Berge, Hisashi Tamaru, Michael Freitag, Eric U. Selker

**Affiliations:** Institute of Molecular Biology, University of Oregon, Eugene, Oregon, United States of America; Medical Research Council Human Genetics Unit, United Kingdom

## Abstract

Eukaryotic genomes are partitioned into active and inactive domains called euchromatin and heterochromatin, respectively. In *Neurospora crassa*, heterochromatin formation requires methylation of histone H3 at lysine 9 (H3K9) by the SET domain protein DIM-5. Heterochromatin protein 1 (HP1) reads this mark and directly recruits the DNA methyltransferase, DIM-2. An ectopic H3 gene carrying a substitution at K9 (*hH3^K9L^ or hH3^K9R^*) causes global loss of DNA methylation in the presence of wild-type *hH3* (*hH3^WT^*). We investigated whether other residues in the N-terminal tail of H3 are important for methylation of DNA and of H3K9. Mutations in the N-terminal tail of H3 were generated and tested for effects *in vitro* and *in vivo*, in the presence or absence of the wild-type allele. Substitutions at K4, K9, T11, G12, G13, K14, K27, S28, and K36 were lethal in the absence of a wild-type allele. In contrast, mutants bearing substitutions of R2, A7, R8, S10, A15, P16, R17, K18, and K23 were viable. The effect of substitutions on DNA methylation were variable; some were recessive and others caused a semi-dominant loss of DNA methylation. Substitutions of R2, A7, R8, S10, T11, G12, G13, K14, and P16 caused partial or complete loss of DNA methylation *in vivo*. Only residues R8-G12 were required for DIM-5 activity *in vitro*. DIM-5 activity was inhibited by dimethylation of H3K4 and by phosphorylation of H3S10, but not by acetylation of H3K14. We conclude that the H3 tail acts as an integrating platform for signals that influence DNA methylation, in part through methylation of H3K9.

## Author Summary

DNA methylation is a common feature of eukaryotic genomes. Methylation is typically associated with silenced chromosomal domains and is essential for development of plants and animals. Although the control of DNA methylation is not well understood, recent findings with model organisms, including the fungus *Neurospora crassa*, revealed connections between modifications of histones and DNA. DNA methylation is dispensable in Neurospora, facilitating genetic studies. Isolation of mutants defective in DNA methylation revealed that a histone H3 methyltransferase, DIM-5, is required for DNA methylation. DIM-5 trimethylates H3K9, which is then recognized by Heterochromatin Protein 1 (HP1), which recruits the DNA methyltransferase DIM-2. We investigated the possibility that H3 provides a platform to integrate information relevant to whether the associated DNA should be methylated. Indeed, we found that DIM-5 is sensitive to methylation of H3K4 and phosphorylation of H3S10. Our analyses further revealed that H3K14 is critical *in vivo*, but not because acetyl-K14 inhibits DIM-5. We also found that H3R2 is essential for DNA methylation *in vivo* but not important for DIM-5 activity. Interestingly, we found H3 mutants that show recessive defects in DNA methylation and others with dominant effects. We also defined a set of H3 mutations that are lethal.

## Introduction

The primary structures of histones, the small basic proteins that are complexed with DNA to form chromatin in eukaryotes, are highly conserved but not invariant [1], [2]. For example, comparisons between a sea urchin and the filamentous fungus *Neurospora crassa* reveal that the two most highly conserved histones, H3 and H4, have 16/135 and 9/102 amino acid differences, respectively [3], [4]. Pioneering studies with the yeasts *Saccharomyces cerevisiae* and *Schizosaccharomyces pombe* revealed that mutation, or even deletion, of some histone residues is not lethal, allowing for genetic studies of structure/function relationships of these prominent proteins [5]–[9].

Histones are subject to a variety of posttranslational modifications including methylation, acetylation, phosphorylation, ubiquitylation, sumoylation, as well as ADP-ribosylation, deimination and proline isomerization [1], [10]. An explosion of findings has implicated these modifications in various fundamental cellular processes, including transcription, alternative splicing, replication, chromosome condensation, recombination, DNA repair and DNA methylation [1], [10], [11]. One approach to investigate potential involvement of modifications of a particular histone residue is to test for effects of substitutions that prevent the modification or mimic the modified state. For example, because acetylation of lysines reduces the positive charge of this residue, it can be informative to test the effect of substituting lysines with non-modifyable residues that are either neutral (e.g. glutamine) or positively charged (e.g. arginine) [12]. To gain insight into residues involved in DNA methylation and other cellular processes, we carried out a systematic analysis of the heavily modified N-terminus of *N. crassa* histone H3 [13].

Neurospora is an excellent organism to study effects of histone H3 mutations in part because the wild-type genome contains a single H3 gene, *hH3* [3], [4]. A direct link between H3 modifications and DNA methylation was originally demonstrated in Neurospora when DNA methylation was found to depend on trimethylation of histone H3 lysine 9 (H3K9me3) [14], [15]. In this organism, DNA methylation is primarily found at sequences that are products of a genome defense mechanism called RIP (Repeat Induced Point mutation), which is triggered by sequence repeats [16], [17]. Duplicated sequences are efficiently detected and mutated during the sexual phase of the Neurospora life cycle in the period between fertilization and nuclear fusion. Both copies of duplicated sequences are peppered with numerous C:G to T:A mutations, rendering the resulting sequences AT-rich, and the remaining cytosines are typically methylated [18], [19]. A single DNA methyltransferase (DIM-2), guided by Heterochromatin Protein 1 (HP1) [20], [21], is responsible for all known DNA methylation in Neurospora [22]. HP1 binds to H3K9me2 and even more tightly to H3K9me3, the predominant form found in Neurospora [23], [24]. Mutation of the *dim-5* gene, which encodes an H3K9 methyltransferase responsible for most if not all H3K9me3 in Neurospora, results in complete loss of DNA methylation [14]. DIM-5 is part of the DCDC (DIM-5/-7/-9/Cul4/DDB1 Complex), and depends on DIM-7 for recruitment to future heterochromatin domains [25]–[27]. All components of the DCDC are required for normal H3K9 methylation [26]. It is also known that methylation of some regions requires dephosphorylation of H3S10 by PP1 [28]. The current study was carried out to decipher which residues of H3 play a role in DNA methylation. We expected to identify interactions with DIM-5 as well as interactions with other components of the methylation machinery, such as HP1. In plants [29]–[31] and animals [32], H3K9 methylation also directs some DNA methylation, raising the possibility that our findings will provide insights into shared mechanisms operating in a variety of organisms.

Initial experiments demonstrated that introduction of an ectopic H3 gene bearing substitutions at lysine 9 (e.g. *hH3^K9R^*) can cause dominant loss of DNA methylation and can reactivate a methylation-silenced transgene, *hph^me^* [14]. Structural studies demonstrated intimate contacts between the H3 tail (residues A7-G12) and the DIM-5 backbone [33], suggesting that additional residues may be important for DIM-5 activity and DNA methylation. We wished to test both for possible dominant effects, as initially detected for K9 substitutions [14], and for possible recessive effects on DNA methylation, H3K9me and DIM-5 activity. We therefore first transformed a strain harboring the methylation-silenced transgene (*hph^me^*) to screen for substitutions that cause a dominant loss of DNA methylation. We next constructed substitution strains to reveal recessive mutations. Our current results suggest that multiple H3 tail residues, or their modifications, are involved in DNA methylation. We found both recessive and dominant effects.

## Results

### Amino-acid substitutions in the N-terminal tail of H3 reactivate a transgene and reduce DNA methylation

**Figure 1:**
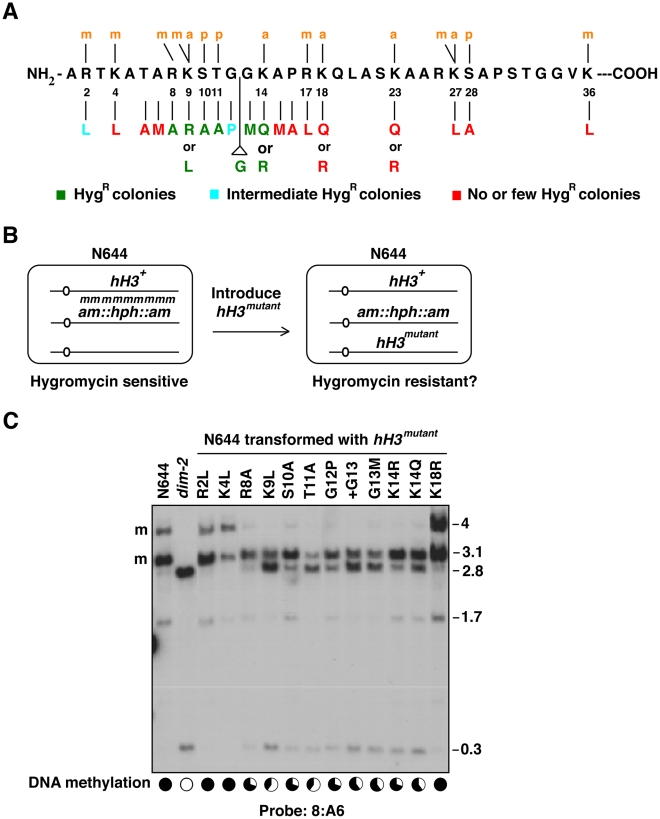
Reactivation of a methylation-silenced transgene by amino acid substitutions in the N-terminal tail of histone H3. (A) Sequence of *N. crassa* H3 amino-terminal tail and summary of results after transformation of strain N644 with *hH3* alleles bearing indicated mutations. (See also Figure S1). (B) Scheme to test the possible role of H3 residues in DNA methylation. Strain N644 harbors a methylated, silenced *hph* transgene, which confers resistance to hygromycin when it is expressed. Transformation of this strain with mutant forms of *hH3* normally result in a mixture of wild-type and mutant histone in the cell. If the tested amino acid residue is critical for DNA methylation this may cause a semi-dominant loss of DNA methylation and thus expression of *hph*. (C) Partial loss of DNA methylation in transformants with native *hH3* plus ectopic mutant *hH3* alleles. DNA from selected hygromycin-resistant transformants [*hH3^R2L^* (N3079); *hH3^K4L^* (N3081); *hH3^R8A^* (N3082); *hH3^K9L^* (N3084); *hH3^S10A^* (N3085); *hH3^T11A^* (N3087); *hH3^G12P^* (N3088); *hH3^+G13^* (N3090); *hH3^G13M^* (N3092); *hH3^K14R^* (N3094); *hH3^K14Q^* (N3096); *hH3^K18R^* (N3098)] was digested with 5-methylcytosine-sensitive restriction enzymes (*Bam*HI and *Eco*RI) and analyzed by Southern hybridization. The blots were probed for the 8:A6 region [18], which is normally methylated (m), giving rise to 4.0 and 3.1 kb fragments. Complete loss of methylation, as in the *dim-2* mutant, gives rise to 2.8 and 0.3 kb fragments. The levels of DNA methylation in various strains, determined by the relative intensities of the 3.1 and 2.8 kb bands using ImageJ [79] are indicated in the pie-charts (bottom). To convert the images into the approximate methylation levels conveyed in the pie graphs, we used densitometry to compare the intensity of bands representing methylated and unmethylated sites and normalized the results to that in wild-type (defined as 100%). Similar results were observed for other methylated regions including ψ_63_, 2:B3 (see also Figure S2, Table S4), 5:B8, 8:G3, 1d21 and 8:F10 (data not shown).

Roughly half of the approximately 40 amino acid residues in the highly conserved N-terminal tail of histone H3 are subject to covalent modifications (Figure S1) [1], [2], [10]. To improve our understanding of the role of H3 in DNA methylation, we carried out mutational analyses of these and other residues of potential interest, such as those around lysine 9 (Figure 1A). Acetylatable lysines were changed to arginines (K→R) or to glutamines (K→Q) to simulate the hypoacetylated or acetylated states, respectively. Lysines and arginines that might be methylated were changed to leucines (K/R→L), and potentially phosphorylated serines and threonines were changed to alanines (S/T→A) to prevent modification. We also tested for a constraint on the spacing of residues by inserting an extra glycine at GG12,13 (+G13). We took advantage of a simple genetic test for effects on DNA methylation. Mutant alleles were introduced into a hygromycin-sensitive reporter strain, N644, in which the hygromycin phosphotransferase gene, *hph*, was reversibly silenced by DNA methylation [34]. In our initial experiments, we co-transformed N644 with the mutant constructs along with a dominant selectable marker, *Ben^R^*, which confirms resistance to benomyl [14]. Loss of *hph* methylation caused by dominant or semi-dominant effects of the *hH3^mutant^* alleles would render the strain resistant to hygromycin (Figure 1B). Although co-tranformation is extremely efficient in Neurospora (typically 50–90% of transformants incorporate the non-selected DNA), representative transformants were tested to verify that the H3 construct had been integrated in a fraction of the tranformants that did not produce hygromycin resistant strains (data not shown).

We found that substitutions of any residue between positions 8 to 14 caused hygromycin resistance (Figure 1A, Table 1 and Table S1). Similarly, insertion of an extra glycine at GG12-13 (+G13) caused hygromycin resistance. Transformation experiments were repeated 4–8 times with similar results (Table 1). Some substitutions, notably G12P and R2L, gave variable or weaker hygromycin resistance; transformations with constructs bearing these substitutions only gave rise to hygromycin resistant colonies in ∼50% of the transformations. Overall, phenotypic analyses of transformants suggested that silencing depends on R2 and residues around K9 (R8 to K14) and that the spacing between residues around GG12-13 is important.

**Table 1:** DNA methylation analyses of Hyg^R^ colonies obtained in cotransformations of N644 with *hH3* alleles.

Substitution	Number of transformations	Fraction yielding resistance to hygromycin^1^	Fraction hypomethylated^2^
**R2L**	6	3/6	0/3^3^
**K4L**	6	0/6	-
**T6A**	4	1/4	0/1^4^
**A7M**	4	0/4	-
**R8A**	5	3/5	1/3^5^
**K9L**	8	3/8	3/3
**K9R**	8	3/8	3/3
**S10A**	8	5/8	5/5
**T11A**	6	5/6	5/5
**G12P**	6	3/6	1/3
**+G13**	7	5/7	3/5
**G13M**	5	4/5	2/4
**K14Q**	8	7/8	6/7
**K14R**	8	7/8	7/7
**A15M**	6	1/6	0/1^6^
**P16A**	4	0/4	-
**R17L**	4	1/4	0/1^6^
**K18Q**	6	1/6	0/1^6^
**K18R**	6	2/6	1/2^7^
**K23Q**	6	0/6	-
**K23R**	6	1/6	0/1^6^
**K27L**	6	0/6	-
**S28A**	6	0/6	-
**K36A**	6	0/6	-

1 Fraction transformations that gave rise to HygR colonies.

2 Fraction transformations that showed loss of DNA methylation in Southern blotting.

3 One transformant out of 12 tested exhibited hypomethylation.

4 Of the two HygR transformants obtained, both were methylated.

5 Half of the transformants tested (28) demonstrated loss of DNA methylation.

6 All HygR transformants tested were methylated.

7 Two HygR transformants out of 11 tested were hypomethylated.

The methylated *hph* allele is somewhat unstable; its reversion frequency is ∼10^−5^ [25], [34]. To confirm that H3 mutations caused loss of DNA methylation, we directly examined the methylation status of genomic DNA by Southern hybridization using 5-methylcytosine-sensitive restriction endonucleases. Blots were probed for several genomic regions that are typically methylated [18]. DNA from the parental strain (N644) and from a DNA methyltransferase mutant (*dim-2*) served as positive and negative controls, respectively, and DNA from several independent transformants were analyzed for each *hH3* construct. Consistent reductions in DNA methylation were observed for all transformants with S10A, T11A, G12P, +G13, G13M, K14R, K14Q, K9L and K9R mutations (Figure 1C, Figure S2A and S2B, Table 1 and Table S1). Curiously, about half of the hygromycin-resistant colonies that came from the R8A transformations exhibited loss of DNA methylation. None of the few hygromycin-resistant colonies that were obtained with the T6A, A15M, R17L, K18R, K18Q and K23R constructs showed changes in DNA methylation. In the case of R2L, although hygromycin-resistant colonies were obtained in half of the trials, only one of twelve mutant strains analyzed showed a significant reduction in DNA methylation. This variability might result from differences in copy number and/or chromosomal location of the integrated mutant alleles. Indeed, we found that some hygromycin-resistant transformants had single ectopic integrations of *hH3^mutant^* genes while others had multiple integrations (Figure S2C). Multiple integrations might result in a dose-dependent loss of DNA methylation. Of course, hygromycin resistance may also result from unknown genetic or epigenetic effects. To address these possibilities, we developed a system to construct strains with single copies of mutant alleles inserted at a particular location in the genome, as described below.

### Amino acid residues surrounding K9 are required for DIM-5 activity in vitro

**Figure 2:**
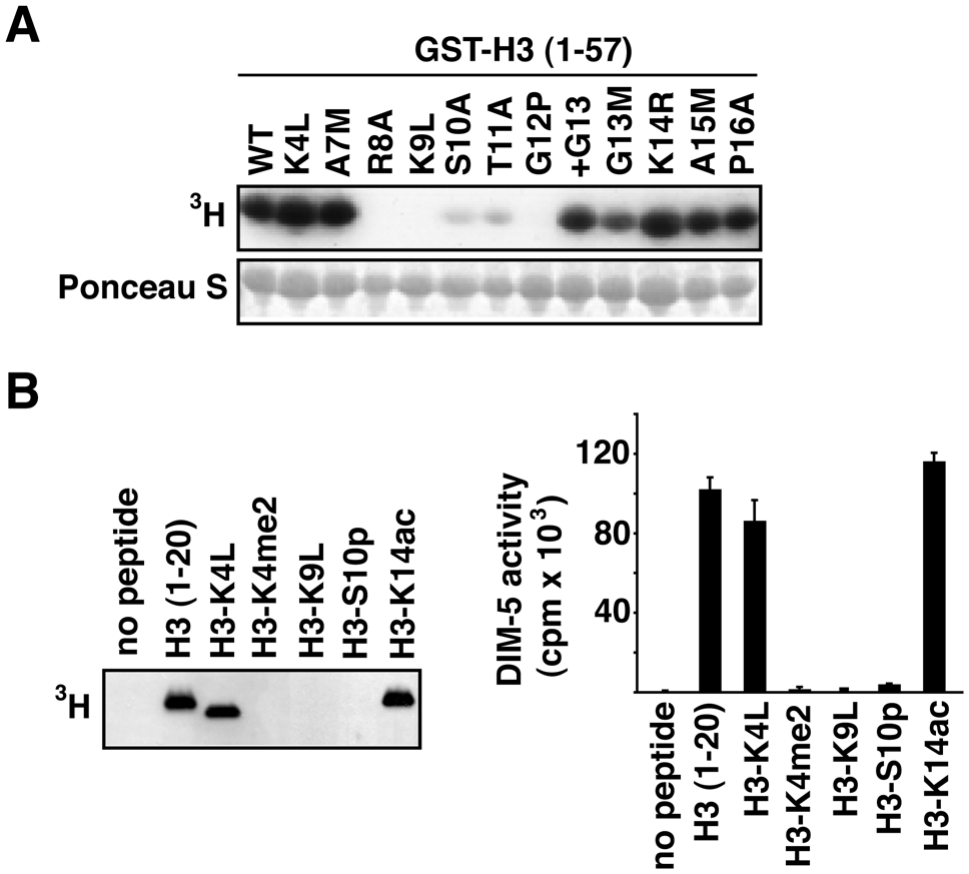
DIM-5 activity on histone H3 peptides. (A) Residues surrounding H3K9 are critical for DIM-5 activity. Histone methyltransferase assays were performed with GST-H3 (1–57) bound to glutathione-agarose and *S*-adenosyl [methyl-^3^H]-L-methionine as the methyl group donor and analyzed by gel electrophoresis and fluorography. The membrane was stained with Ponceau S to confirm amount of protein loaded (bottom panel). (B) DIM-5 is sensitive to modifications of the H3 tail. *In vitro* assays were performed with unmodified H3 peptide (residues 1–20) or with peptides bearing covalent modifications (K4me2, S10ph and K14ac) or a substitution (K4L). Incorporation of the methyl group was analyzed by fluorography (left panel) or by liquid scintillation counting (right panel, each bar represents an average of three reactions).

H3 substitutions may cause loss of DNA methylation through effects on site recognition or catalysis by the histone methyltransferase, DIM-5. Alternatively, H3 substitutions might affect the DNA methylation pathway downstream of H3K9me. In one approach to distinguish between these possibilities, we tested the *in vitro* activity of DIM-5 on various H3 substrates. We constructed modified forms of a GST-H3 (residues 1–57) fusion corresponding to the amino acid replacements that we tested *in vivo*. The fusion proteins were incubated with DIM-5 and radio-labeled methyl-group donor (*S*-adenosyl methionine), fractionated by SDS-polyacrylamide gel electrophoresis and tested for incorporation of ^3^H-methyl groups by fluorography (Figure 2A). As expected from our previous finding that H3K9 is the only target for DIM-5 [14], the K9L substitution abolished DIM-5 activity. The R8A and G12P mutations also completely inhibited DIM-5 activity. The S10A and T11A mutations greatly reduced, but did not abolish, DIM-5 activity. Slightly reduced activity was observed with the G13M protein in most assays performed. In contrast, K4L, A7M, +G13, K14R, A15M and P16A mutations had no significant effect on DIM-5 activity. The K14R mutation was not inhibitory *in vitro* while this change caused striking hygromycin resistance and loss of methylation *in vivo* (Figure 1). We conclude that the effects of mutations in H3 residues 8–12 are likely due to direct effects on DIM-5/H3 interactions while the inhibitory effects on DNA methylation of K14R is presumably due to some unknown downstream effects (e.g. on HP1 or DIM-2 action).

### Methylation of H3K4 and phosphorylation of H3S10, but not acetylation of K14, inhibit methylation of H3K9 by DIM-5

The H3 tail is subject to a variety of post-translational modifications (Figure S1) that could potentially play a role in controlling DIM-5 action. Indeed, there is precedence for effects of modifications on the activity of histone methyltransferases related to DIM-5. In particular, SUV39H1, SETDB1 and Clr4 have been shown to be sensitive to phosphorylation of H3S10 (H3S10ph) [35]–[37]. We were most interested in the possibility that H3K4me, a mark common in euchromatic regions, might interfere with DNA methylation in Neurospora. In addition, we wished to test the effect of acetylation of H3K14 (H3K14ac) because this modification is apparently countered by histone deacetylase 1, which is important for DNA methylation in Neurospora [38], and because substitution of K14 inhibited DNA methylation *in vivo* but did not noticeably affect DIM-5 activity *in vitro* (Figure 1A, 1C and Figure 2A). We therefore tested the effect of methylation of H3K4, acetylation of H3K14 and phosphorylation of H3S10 on *in vitro* DIM-5 activity with peptides covering residues 1–20 of H3 (Figure 2B). As expected, a peptide that had a substitution at the target residue, H3K9, completely abolished DIM-5 activity. Robust activity was found with a H3K14ac peptide. This contrasts results obtained with the H3K9 methyltransferase SETDB1 [37] but is consistent with findings with the SUV39H1 and Clr4 H3K9 methyltransferases [35], [36]. Essentially no DIM-5 activity was detected with H3S10ph or H3K4me2 peptides, whereas a peptide bearing the K4L substitution showed full activity. H3K4me2 did not affect H3K9 methylation by SETDB1 or Clr4 [36], [37]. Our results support the idea that H3K4me2 and H3S10ph impact DNA methylation by preventing DIM-5 activity. In contrast, the importance of H3K14 presumably results from an effect downstream of H3K9 methylation by DIM-5.

### Recessive effects of hH3 mutations

**Figure 3:**
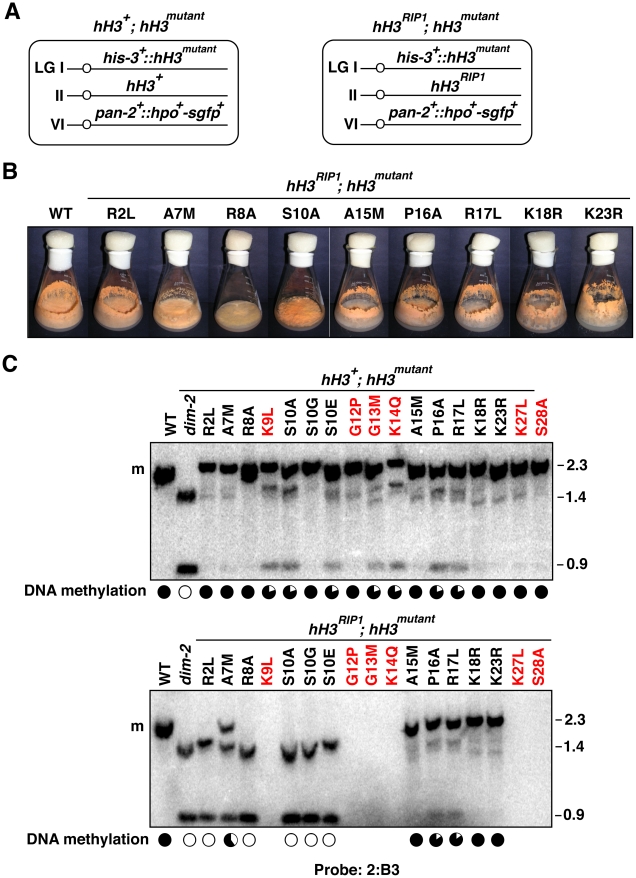
Substitutions in the H3 tail can cause recessive or semi-dominant loss of DNA methylation. (A) Generalized genotypes of strains harboring two copies of *hH3*. Various substitution alleles (*hH3^mutant^*) were integrated at an ectopic location (*his-3*) and combined with either the wild-type allele (*hH3^+^*; left) or the null allele (*hH3^RIP1^*; right) at the native *hH3* locus. A gene encoding HP1-GFP was integrated at the *pan-2* locus to visualize heterochromatin by microscopy (Figure 4B). (B) Defective asexual development in stains containing mutant H3. The H3 mutants showed reduced growth relative to wild-type (N150) and reduced production of asexual spores. The strains tested carried the following mutations: *hH3^R2L^* (N3520); *hH3^A7M^* (N3537); *hH3^R8A^* (N3542); *hH3^S10A^* (N3474); *hH3^A15M^* (N3553); *hH3^P16A^* (N3556); *hH3^R17L^* (N3560); *hH3^K18R^* (N3565); *hH3^K23R^* (N3568). All strains were grown on solidified minimal medium with 1.5% sucrose for 7 days at 32°C. (See also Figure S3). (C) Loss of DNA methylation caused by H3 substitutions. Southern blotting with DNA isolated from strains that contain either a mixture of wild-type and altered H3 [top panel: *hH3^R2L^* (N3524); *hH3^A7M^* (N3540); *hH3^R8A^* (N3543); *hH3^K9L^* (N3544); *hH3^S10A^* (N3494); *hH3^S10G^* (N3475); *hH3^S10E^* (N3476); *hH3^G12P^* (N3546); *hH3^G13M^* (N3548); *hH3^K14Q^* (N3550); *hH3^A15M^* (N3554); *hH3^P16A^* (N3558); *hH3^R17L^* (N3562); *hH3^K18R^* (N3566); *hH3^K23R^* (N3570); *hH3^K27L^* (N3572); *hH3^S28A^* (N3573)] or just altered H3 [bottom panel: *hH3^R2L^* (N3520); *hH3^A7M^* (N3537); *hH3^R8A^* (N3542); *hH3^S10A^* (N3474); *hH3^S10G^* (N3477); *hH3^S10E^* (N3478); *hH3^A15M^* (N3553); *hH3^P16A^* (N3556); *hH3^R17L^* (N3560); *hH3^K18R^* (N3565); *hH3^K23R^* (N3568)]. DNA was digested with the 5mC-sensitive restriction enzyme *Ava*II and the blots were probed for the methylated region 2:B3 [18]. Levels of DNA methylation are summarized as in Figure 1 based on the relative intensitites of 2.3 and 0.9 kb bands. Similar results were observed for other methylated regions including 2:G12 and 8:A6 (see also Figures S4 and S5, Table S4).

In theory, Neurospora transformants of strain N644 may have resulted either from ectopic insertion of *hH3^mutant^* genes or from gene replacement events. In practice, only ectopic insertions were obtained, and most transformants had single integrations (Figure S2C), which is unusual for transformation of Neurospora. This raised the possibility that transformants bearing exclusively, or even predominantly, altered H3 alleles may not be viable. To test this possibility, and to investigate if some H3 substitutions might be recessive and thus show defects that were masked by the simultaneous presence of wild-type H3, we developed a system to test *hH3* constructs as single copies at a defined genomic location, in the presence or absence of a wild-type allele. The system takes advantage of a non-functional *hH3* allele at the endogenous locus (*hH3^RIP1^*) that we generated by RIP when we crossed two strains homozygous for an *hH3^S10E^* allele at the *his-3* locus [28]. The resulting *hH3^RIP1^* strain with *hH3^S10E^* at *his-3* was crossed with transformants bearing other *hH3* substitution alleles integrated at *his-3* to test whether progeny with substitution alleles at *his-3* would be viable in the absence of a functional allele (Figure 3A; Text S1). For most of our substitutions, including those at R2, A7, R8, S10, A15, P16, R17, K18 and K23, we were able to build such (*hH3^RIP1^; hH3^mutant^*) strains. Thus the entire cellular pool of H3 carries the relevant substitution in these strains. Although viable, all these strains showed defects in vegetative development (Figure 3B, Figure S3) and were female sterile (data not shown). For substitutions of other residues (K9, G12, G13, K14, K27, S28 and K36), we were only able to obtain strains with the substitution allele at *his-3* when the wild-type allele was present at the native locus (*hH3^WT^; hH3^mutant^*), suggesting that these mutations are lethal in the absence of wild-type H3. We also found evidence that substitutions of K4 and T11 are not tolerated in the absence of a wild-type allele (data not shown). Although it was conceivable that some or all of these mutations simply affected protein stability, their semi-dominant effects argued against this.

Analyses of DNA methylation in the targeted H3 mutant strains was informative (Figure 3). Where comparisons could be made with results obtained with the strains bearing one or more copies of mutant constructs at undefined sites, the strains with precisely one wild-type and one mutant H3 gene gave similar results (Figure 3C and Figure S4; Table S4). For example, mutation of K14 gave equivalent results with both experimental schemes. [Incidentally, it is noteworthy that the extent of loss of DNA methylation caused by substitutions in K14 was similar to that caused by substitutions in K9 even though the K14 mutations, unlike the K9 mutations, did not substantially interfere with methylation by DIM-5 *in vitro* (Figure 2) or *in vivo* (data not shown)]. We did observe some variability between experiments and among methylated regions tested (Table S4). R8A, which caused a partial (∼25%) reduction in DNA methylation in cotransformation experiments (Figure 1 and Figure S2), showed comparable reduced methylation at the 2:G12 region when the mutant allele was at the *his-3* locus but did not show significant loss of methylation at 8:A6 or 2:B3. The K9L mutation, which resulted in clear loss of methylation in cotransformants at all regions tested also showed obvious reduced methylation at 8:A6 and 2:B3 but not at 2:G12 when targeted to *his-3*. Substitutions of S10 with A or E resulted in partial loss of DNA methylation at all sites examined but S10G did not cause loss of methylation at any region tested, when there was a wild-type H3 gene in the same strain. Mutations of R2, A7, A15, K18, K23, K27 and S28, which did not reveal loss of silencing in the cotransformation experiments also showed no loss when the mutant allele was targeted to *his-3* in the presence of the native H3 gene. Mutations of P16 and R17, which also showed no effect in the cotransformation experiments, caused modest reduction of DNA methylation, but only in the 2:B3 region. In contrast, substitution of residues G12 showed significant loss of methylation in the contransformation experiments but not in the targeted transformants and mutation of G13 showed a modest reduction of methylation in the latter experiments and a greater reduction in the contransformants.

**Figure 4:**
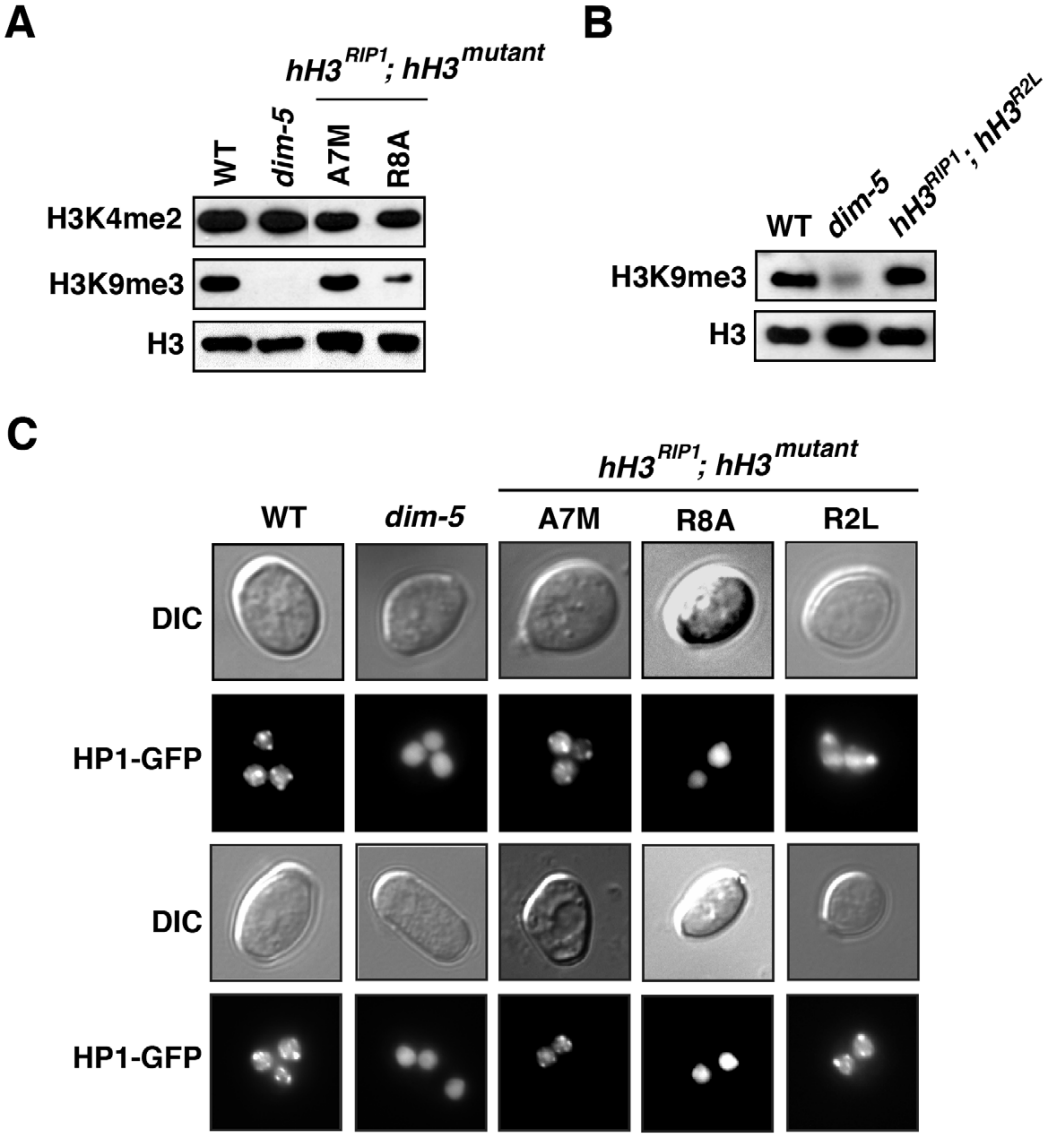
Reduced H3K9 methylation and HP1 binding in selected *hH3* substitution strains. (A) Reduction in H3K9 methylation in the A7M and R8A substitution strains. Western blots of nuclear extracts from wild-type (N150), *dim-5* (N2264), *hH3^A7M^* (N3537) and *hH3^R8A^* (N3542) strains. An antibody recognizing the C-terminus of H3 served as a loading control. (B) Apparently normal H3K9 methylation and HP1 binding in the *hH3^R2L^* mutant. Western blots of nuclear extracts from wild-type (N150), *dim-5* (N2264) and *hH3^R2L^* (N3520) strains. An antibody recognizing the C-terminus of H3 served as a loading control. (C) Localization of HP1-GFP in H3 substitution strains. Duplicate sets of images generated by light (DIC) and fluorescence (HP1-GFP) microscopy on germinating conidia of wild type (N2534), *dim-5* (N2542), *hH3^A7M^* (N3537), *hH3^R8A^* (N3542) and *hH3^R2L^* (N3520) strains are shown.

The results summarized above unequivically indicated that a number of mutations of the H3 gene are semi-dominant. Interestingly, we also found several mutant constructs that showed dramatic effects on DNA methylation only when they provided all of the H3, i.e. they were recessive. The most striking results were obtained for the R2L mutation. Although this change showed little, if any, effect in the presence of normal H3 (Figure 1 and Figure 3), the mutant allele, when alone, caused a nearly complete loss of DNA methylation (Figure 3C, Figures S4 and S5) Interestingly, methylation of H3 K9 and HP1 binding appeared normal (Figure 4), suggesting that R2 plays a role downstream of these events.

The A7M and R8A substitution alleles also showed marked reductions in DNA methylation alone, but little or no reduction when present along with the wild-type allele (Figure 3C). Of these two mutants, R8A gave the stronger effect, consistent with its marked inhibition of H3K9 methylation *in vitro* (Figure 2). We confirmed that the R8A substitution strain, but not the A7M strain, also shows greatly reduced H3K9me3 and mislocalization of HP1 *in vivo* (Figure 4).

## Discussion

We took advantage of the single-copy status of the histone H3 gene in Neurospora to explore the possible involvement of particular amino acid residues of H3 in DNA methylation. The study was facilitated by a methylated *hph* allele that allowed detection of partial loss of DNA methylation. Previous work revealed that transformation of the reporter strain with alleles of *hH3* bearing substitutions in H3K9 caused almost complete loss of DNA methylation, while retaining a wild-type allele of *hH3*, i.e. the mutations were semi-dominant, as if the altered H3 “poisoned” the methylation machinery [14]. This may reflect a requirement for the HP1 dimer to bind pairs of methylated H3K9 residues within one nucleosome or between adjacent nucleosomes [39]. Failure to isolate transformants bearing multiple copies of the mutant allele further suggested that a preponderance of H3 with this substitution might not be tolerated by Neurospora [14].

Here we explored the generality of these observations by extending our analysis to include all residues in the N-terminal tail that are thought to be subject to post-translational modification [1], [10]. In addition to testing various residues with our system pioneered for K9, we also developed a scheme to test directly for viability of strains bearing mutant alleles in the absence of a wild-type copy of *hH3* [28]. This approach identified a number of residues that are essential for viability and revealed nonlethal mutations that resulted in recessive DNA methylation defects (Table 2). The most striking recessive defects were due to R2L, A7M, R8A and S10G substitutions. S10A and S10E showed semi-dominant defects that became much more pronounced in the absence of the wild-type allele while some other changes, including P16A and R17L, showed modest defects either in the presence or absence of the wild-type allele. Most, but not all, residues identified as important for DNA methylation *in vivo* were also important for DIM-5 histone methyltransferase activity *in vitro*. In particular, R8, K9, S10, T11, G12 and G13 were required for H3K9me3 by DIM-5 *in vitro*, while substitutions in every residue from A7 through R17 caused loss of DNA methylation *in vivo*. We also found that the spacing between residues can be important since introduction of an extra glycine at GG12-13 caused a loss of DNA methylation. Finally, we found that DIM-5 is sensitive to certain H3 modifications (H3K4me2 and H3S10ph) but not to others (H3K14ac).

**Table 2:** Summary of H3 mutagenesis results.

Residue	Essential	Substitution/Insertion	Reactivation of *hph* ^m^	DNA methylation	DIM-5 activity
R2	No	R2L	Yes	− R^c^	+^a^
K4	Yes	K4L	No	+	+
A7	No	A7M	Yes	+/− R	−b
R8	No	R8A	Yes	− S^d^	−
K9	Yes	K9L	Yes	− S	−
S10	No	S10A	Yes	− S	−
		S10E	n.d.	− S	−
		S10G	n.d.	− R	−
T11	Yes	T11A	Yes	− S	−
G12	Yes	G12P	Yes	− S	−
G13	Yes	+G13	Yes	− S	−
G13	Yes	G13M	Yes	− S	−
K14	Yes	K14R	Yes	− S	+
A15	No	A15M	No	+	+
P16	No	P16A	No	− S	+
R17	No	R17L	No	− S	+
K18	No	K18R	No	+	+
K23	No	K23R	No	+	+
K27	Yes	K27L	No	+	+
S28	Yes	S28A	No	+	+
K36	Yes	K36L	No	+	+

a Normal wild-type levels.

b Major reduction or loss.

c Recessive loss of DNA methylation.

d Semi-dominant loss of DNA methylation.

n.d. not determined.

Our results provide clues to the role of histone H3 in the control of DNA methylation. No comparable study has been carried out in a system with DNA methylation. Extensive mutational analyses of histones have been carried out in the budding yeast *S. cerevisiae* [6]–[8], however, and more limited studies have been carried out in the fission yeast *S. pombe* [9], which unlike *S. cerevisiae*, sports H3K9 methylation. We found that point mutations in K4, K9, T11, G12, G13, K14, K27 and S28 could not be tolerated by Neurospora in the absence of a normal allele. Interestingly, unlike yeasts that have been examined, K27 is subject to methylation in Neurospora, but this modification is not essential (K. Jamieson and E. Selker, unpublished observation). Substitutions in K4, K9, S10 and K14 are tolerated in fission yeast [9], [40] although they do interfere with heterochromatin function, as in Neurospora. A curious result from studies in budding yeast is that effects of histone mutations appear to depend to some extent on strain background [7]. Our observation that a mutant allele containing an extra residue (+G13) is tolerated only in presence of a sheltering wild-type allele implies that the proper spacing between H3 N-terminal tail residues is critical for viability. This provides a possible explanation for the observed lethality of a deletion of residues 4 through 10 in budding yeast, even though substitutions in individual residues from 1 through 39 were tolerated [7].

Phosphorylation of T3, T6, S10, T11 and S28 in the H3 tail has been found to be required for chromatin-dependent processes including gene silencing in several systems [28], [41]–[47]. Based on the crystal structure of DIM-5, the presence of a phosphate on H3S10 should prevent the interactions between the hydroxyl group of S10 and residues in DIM-5 that are essential for optimal catalytic activity (Y283 and D209) [33]. The expected importance of the dephosphorylated state for H3K9 methylation by DIM-5 was supported by findings with strains deficient in the H3S10 phosphatase PP1; an increase in global levels of H3S10ph caused reduced levels H3K9me3 in regions of the genome that loose DNA methylation [28]. This was supported by the observed complete loss of H3K9me and DNA methylation in *hH3^S10A^*, *hH3^S10G^*, *hH3^S10E^* substitution strains (Figure 3C, Figures S4 and S5 and [28]). Information from other systems suggests that phosphorylation of the neighboring residue, T11, may be also important. H3T11ph is associated with chromosome condensation during mitosis and meiosis in plants [48]. In mammals, phosphorylation of H3T11 by PRK1 in response to androgen-dependent stimulation accelerates demethylation of H3K9me3 by the JMJD2C demethylase and activates transcription of target genes [43]. Protein kinase Chk1 also phosphorylates H3T11 and its dissociation from chromatin in response to DNA damage causes a decrease in H3T11ph and release of the H3K9 acetyltransferase GCN5, leading to transcriptional repression [42]. We found that replacement of this residue with alanine caused a semi-dominant loss of DNA methylation (Figure 1C and Figure S2). The main chain carbonyl group of H3T11 forms a hydrogen bond with the DIM-5 backbone (Q285) and the alanine substitution would disrupt this interaction, presumably resulting in the observed loss of DIM-5 activity *in vitro* (Figure 2A and [33]). H3T11ph, like H3S10ph, may impact H3K9 methylation and DNA methylation

It is interesting that substitutions of H3K14 caused a semi-dominant loss of silencing and DNA methylation, especially considering that neither mutation nor acetylation of this residue appreciably affected DIM-5 activity *in vitro* (Figure 2; [49]). Similarly, mammalian SUV39H1 and fission yeast Clr4 are not affected by H3K14ac [35], [36]. One possibility is that H3K14 is important for an activity downstream of methylation of H3K9 by DIM-5. In Neurospora, H3K9me3 is recognized by HP1, which then directly recruits the DNA methyltransferase DIM-2 to the heterochromatin domains [20], [21]. The fact that the H3K14 mutations can only be studied while effectively heterozygous confounded attempts to determine whether HP1 binding is reduced but it is interesting that we found little if any change in H3K9 methylation and HP1 binding in the K14Q mutant (Figure S6). It is noteworthy, that acetylation of this K14 does not affect binding of the *S. pombe* HP1 homologue, Swi6 [50], *in vitro* while mutation of H3K14 in *S. pombe* causes mislocalization of Swi6 [9]. Alhough H3K14ac does not affect H3K9 methyltransferase activity *in vitro*, it remains possible that it interferes with the establishment of H3K9 methylation *in vivo*. In both *S. pombe* and *N. crassa*, the H3K9 methyltransferases are components of multi-protein complexes, the CLRC and DCDC, respectively [25], [26], [51], [52]. Although there are important mechanistic differences between these two complexes, K14 mutations may influence H3 interaction with proteins that guide the H3K9 methyltransferases. The possible importance of H3K14ac in regulation of heterochromatin formation is consistent with the observed requirement of histone deacetylases (HDACs) for DNA methylation in *N. crassa* and for heterochromatin formation in *S. pombe* [38], [50], [53]–[55].

An important new finding from our study is that mutation of H3R2 disrupted DNA methylation. Information from other organisms suggests that this arginine is one of several in H3 that are subject to methylation, namely R2, R8, R17 and R26 [56]. Thus it will be of interest to determine if methylation of one or more of the arginines in the H3 N-terminal tail (Figure S1) impact methylation of H3K9 and DNA. Substitutions of both R8 and R17 (*hH3^R8A^* and *hH3^R17L^*) caused semi-dominant loss of DNA methylation, whereas substitution of R2 (*hH3^R2L^*) caused recessive loss of DNA methylation (Figure 3C, Figures S4 and S5). Presumably this reflects different roles of these residues in heterochromatin formation. The structure of DIM-5 complexed with an H3 peptide shows intimate contacts between R8 and the DIM-5 backbone [33]. Methylation of R8 is expected to prevent these interactions, consistent with the lack of DIM-5 activity *in vitro* on a substrate with the R8A substitution (Figure 2A and [49]) and with the observations on strains bearing this substitution, namely reduced H3K9me3, mislocalization of HP1 and loss of DNA methylation (Figure 4A and 4B). In contrast to the situation with R8, R2 lies outside of the region bound by DIM-5 (A7-G12) [33] and substitutions in R2 have little effect on DIM-5 activity *in vitro* [49]. Conceivably, the loss of DNA methylation observed in the R2L mutant is mediated by H3K4 methylation. It is known that H3R2me2 indirectly blocks H3K4me3 in both budding yeast and mammals [57]–[59]. Though H3K4me2 inhibits DIM-5 activity *in vitro* (Figure 2B and 2C), we observed wild-type levels of H3K9 methylation in the R2L mutant *in vivo* and apparently normal localization of HP1 (Figure 4). These observations suggests that the recessive loss of DNA methylation caused by R2L substitution results from disruption of a step downstream of H3K9 methylation and HP1 binding.

Our finding that DIM-5 activity is inhibited by methylation of H3K4 (Figure 2B) raises the possibility that effects on DNA methylation may reflect effects on methylation of H3K4. Indeed, methylated forms of H3K4 and H3K9 appear mutually exclusive in the Neurospora genome [19]. The mammalian H3K9 methyltransferase G9a is similarly sensitive to methylated forms of K4 [60] but, surprisingly, other H3K9 HMTases including *S. pombe* Clr4, mammalian SETDB1 and dSU(VAR)3-9, have been reported to methylate H3 peptides bearing K4me2 [35], [36], [61]. The establishment of H3K9me in these organisms apparently requires the concerted activity of various H3K4 demethylases, specifically SU(VAR)3-3 in *Drosophila* and Lid2 in fission yeast [62], [63]. In mammals, the DNA methyltransferase regulator DNMT3L directly interacts with the H3 tail and this interaction is abolished by methylation of H3K4me [64]. Additionally, Cfp1, a component of the H3K4 methyltransferase complex Setd1, binds unmethylated CpGs and helps establish H3K4me3 domains free of DNA methylation [65], [66].

In summary, our observations in Neurospora suggest that H3 amino-terminal tail residues and their covalent modifications regulate methylation of H3K9, binding of HP1, and one or more downstream events required for the establishment, maintenance and propagation of DNA methylation. Strains generated in this study should be useful to elucidate the role of H3 in other processes in Neurospora including the gene silencing mechanisms, RIP, meiotic silencing and quelling, as well as in other chromatin-based processes including transcription, DNA repair, recombination and the genomic arrangement of histone variants.

## Materials and Methods

### Neurospora strains and methods

Strains used in this study are listed in Table S2. Standard procedures were followed to grow strains and to perform crosses [67]–[69]. Primers used in this study are listed in Table S3.

### Isolation of genomic DNA and Southern analyses

Genomic DNA was prepared as described previously [70], [71] from strains grown with shaking in Vogel's medium N with appropriate supplements at 32°C for 3 days. For Southern blotting, approximately 1 µg DNA was digested overnight with 3–5 units of the desired restriction enzyme, fractionated on 0.8 or 1.0% agarose gels and transferred to nylon membrane [72]. Hybridizations were performed with probes prepared by random hexamer priming [73], as previously described [74].

### Histone H3 methyltransferase assay

DIM-5 assays were carried out in a volume of 20 µl at 10°C for 1 h with 5 µg of the appropriate recombinant GST-H3 variant (H3 residues 1–57) and 2.75 µCi of *S*-adenosyl [methyl-^3^H]-L-methionine (NEN), as previously described [14]. The reaction products were fractionated on SDS-PAGE gel (16%, acrylamide/bis, 29∶1) and incorporation of the radioactive methyl group was analyzed by fluorgraphy using ENTENSIFY (DuPoint). To test the effect of covalent H3 modifications or amino acid substitutions on DIM-5 activity, similar assays were performed with 0.5 µg peptide substrates (modified or unmodified H3 residues 1–20) [15]. The reaction products were analyzed by fluorography or precipitation with 20% TCA, filtration (Millipore GF/F filter), washing and liquid scintillation counting [75].

### Western blotting

Nuclei were isolated as previously described [76], but with minor modifications. The following enzyme inhibitors were added to all buffers: 1 mM sodium butyrate, 1 µM Trichostatin A, 1 µM PMSF, 3 mM DTT, 10 mM sodium fluoride, 1 mM sodium vanadate, 0.1% phosphatase inhibitor cocktail (Sigma, P2850) and 1 µM each of leupepsin, pepstatin and E-64. Western blotting was performed with ∼100 µg of nuclear protein as described previously [15]. The following antibodies were used: α-H3K4me2 (Upstate, 07-030), α-H3K9me3 [15] and α-H3 C-terminal (Active Motif/LP Bio, AR-0144-200). Modifications on histones are represented according to the nomenclature proposed by Turner [77]. All antibodies were used at a dilution of 1∶5000. Horseradish peroxidase (HRP)-conjugated goat antibody against rabbit IgG was used to detect antibody-peptide complexes by chemiluminescene (Thermo Fischer Scientific Inc, USA).

### Microscopy

Dilute suspensions of vegetative spores (conidia) were germinated on solidified Vogel's N medium with 1.5% sucrose and the required supplements for 2 hrs at 30°C. Square pieces of agar with the germinating conidia were cut out from plates and placed on glass slides. Agar pieces were flooded with a few drops of liquid Vogel's N medium and then overlayed with coverslips [78]. Bright-field and fluorescence images were collected on a Zeiss Axioplan 2 Imaging System with a EBQ 100 isolated light source, Endow GFP (S65T) filter (excitation 470, emission 525) and Plan-APOCHROMAT 100×/1.46 N.A. objective. Images were processed with Axiovision (version 4.6.3) and Adobe Photoshop CS (version 8) software.

## Supporting Information

Figure S1 H3 amino-terminal tail residues are highly conserved. Alignment of H3 amino terminal tail from various eukaryotes including *Emericella nidulans* (gi 296337), *Ajellomyces capsulatus* (gi 9624455), *Schizosacchromyces pombe* (gi 5531473), *Neurospora crassa* (gi 18307450), *Saccharomyces cerevisiae* (gi 7019764), *Candida albicans* (gi 68490295), *Magnaporthe grisea* (gi 156630832), *Drosophila melanogaster* (gi 46397771), *Mus musculus* (gi 387198), *Homo sapiens* (gi 1568561), *Caenorhabditis elegans* (gi 12276045), *Arabidopsis thaliana* (gi 4490755). Variable residues are highlighted in green. Covalent modifications including methylation of K and R, acetylation of K and phosphorylation of S and T are highly conserved [10]. Recent studies have also reported proline isomerzation and clipping of H3 tail after A21 [80], [81].
(TIF)

Figure S2 Southern analyses of hygromycin-resistant transformants containing mutant *hH3* at an ectopic location and the wild-type gene at the native locus. Loss of DNA methylation in ψ_63_ (A) and 2:B3 (B) regions. DNA from selected hygromycin-resistant transformants was digested with methylation-sensitive restriction enzymes (*Bam*HI and *Eco*RI for A; *Ava*II for B) and used for Southern hybridizations. The blots were probed for ψ_63_ [18]. Loss of DNA methylation was roughly quantified (pie graphs under autoradiograms) by measuring the ratio of the primary methylation (m) bands and the primary band representing unmethylated DNA (e.g. ratio of 6.4 and 2.7 kb bands for A). The strains are those described in Figure 1C. (C) Determination of mutant *hH3* copies in selected transformants. DNA was digested with *Bam*HI and *Eco*RI and used for Southern hybridizaitons probing with a fragment of the wild-type *hH3* gene.
(TIF)

Figure S3 Poor growth of some H3 substitution strains. Wild type (N150) and strains with single copies of altered H3 genes [*hH3^R2L^* (N3517 and N3520), *hH3^A7M^* (N3530 and N3531), *hH3^R8A^* (N3542), *hH3^S10A^* (N3474 and N3481), *hH3^A15M^* (N3552 and N3553), *hH3^P16A^* (N3556 and N3557), *hH3^R19L^* (N3560 and N3561), *hH3^K18R^* (N3564 and N3565), *hH3^K23R^* (N3568 and N3569)] were grown on solid Vogel's N medium containing 1.5% sucrose in race tubes at 32°C [69]. Growth for each strain is an average of measurements in two tubes.
(TIF)

Figure S4 Loss of DNA methylation caused by H3 substitutions. DNA was digested with the 5mC-sensitive restriction enzyme *Ava*II (panel A) or *Bam*HI and *Eco*RI (panel B) and used for Southern hybridizaitons probing with the indicated regions [18]. Strains are the same as listed in Figure 3C. Loss of DNA methylation was roughly quantified (pie graphs under autoradiograms) by measuring the ratio of the intensities of a methylation (m) band and the primary band representing unmethylated DNA (2.2 and 1.6 kb bands for 2:G12 region; 1.7 and 0.3 kb bands for the 8:A6 region).
(TIF)

Figure S5 Comparison of a strain that contains just mutant H3 with a strain that contains a mixture of wild-type and mutant H3 proteins. A strain containing a mixture of wild-type and mutant H3 [*hH3^WT^; hH3^S10A^* (N3494), indicated in green] grows (panels A & B) and conidiates (panel A) better than a strain that contains just mutant H3 [*hH3^RIP1^; hH3^S10A^* (N3474); indicated in red]. All strains were grown on solid Vogel's N medium containing 1.5% sucrose for 7 days at 32°C. For the race tube measurements (B), results for wild-type (N150; black) and for two strains of each genotype [*hH3^RIP1^; hH3^S10A^* (N3474 and N3481) and *hH3^WT^; hH3^S10A^* (N3494 and N3495)] are shown. Growth for each strain is an average of measurements in two tubes. (C) Southern analysis of genomic DNA isolated from wild type (N150), *dim-2* (N1860), a strain with both wild-type and mutant H3 [*hH3^WT^; hH3^S10A^* (N3494)], and a strain with only mutant H3 [*hH3^RIP1^; hH3^S10A^* (N3474)]. DNA was digested with the 5mC-sensitive restriction enzyme *Sau*3AI and 5mC-insensitive restriction enzyme *Dpn*II and blots were probed for the methylated regions 8:A6, 8:G3 and 9:E1 [18].
(TIF)

Figure S6 The heterozygous H3K14Q substitution mutant shows only slight reductions of H3K9me3 and HP1 in a genomic region that has reduced DNA methyation. (A) Chromatin IP (ChIP) was performed with wild-type (N150), *hH3^S10A^* (N3480) and *hH3^K14Q^* (N3550 and N3551) strains using antibodies to immunoprecipitate H3K9me3 or HP1-GFP. Three independent reactions on two biological replicas are shown. Primer pairs were used in duplex PCRs to amplify an unmethylated region (*hH4-1*) and a methylated region (8:A6) [15], [21]. The numbers indicate the relative enrichment of H3K9me3 or HP1-GFP at 8:A6 compared to *hH4-1*. (B) Quantitation of H3K9me3 levels and HP1 binding using data obtained from three independent duplex PCR reactions.
(TIF)

Table S1 Number of Hyg^R^ colonies obtained in transformations with *hH3* alleles.
(DOCX)

Table S2 Strains used in this study.
(DOCX)

Table S3 Primers used in this study.
(DOCX)

Table S4 Summary of DNA methylation analyses for strains containing wild-type and mutant histone H3.
(DOCX)

Text S1 Supplementary Experimental Methods.
(DOCX)
