# Supplementary figures and images for: Substitutions in the Amino-Terminal Tail of Neurospora Histone H3 Have Varied Effects on DNA Methylation
Source: PLoS Genet. 2011 Dec 29;7(12):e1002423. doi: 10.1371/journal.pgen.1002423 (PMC3248561; doi:10.1371/journal.pgen.1002423)

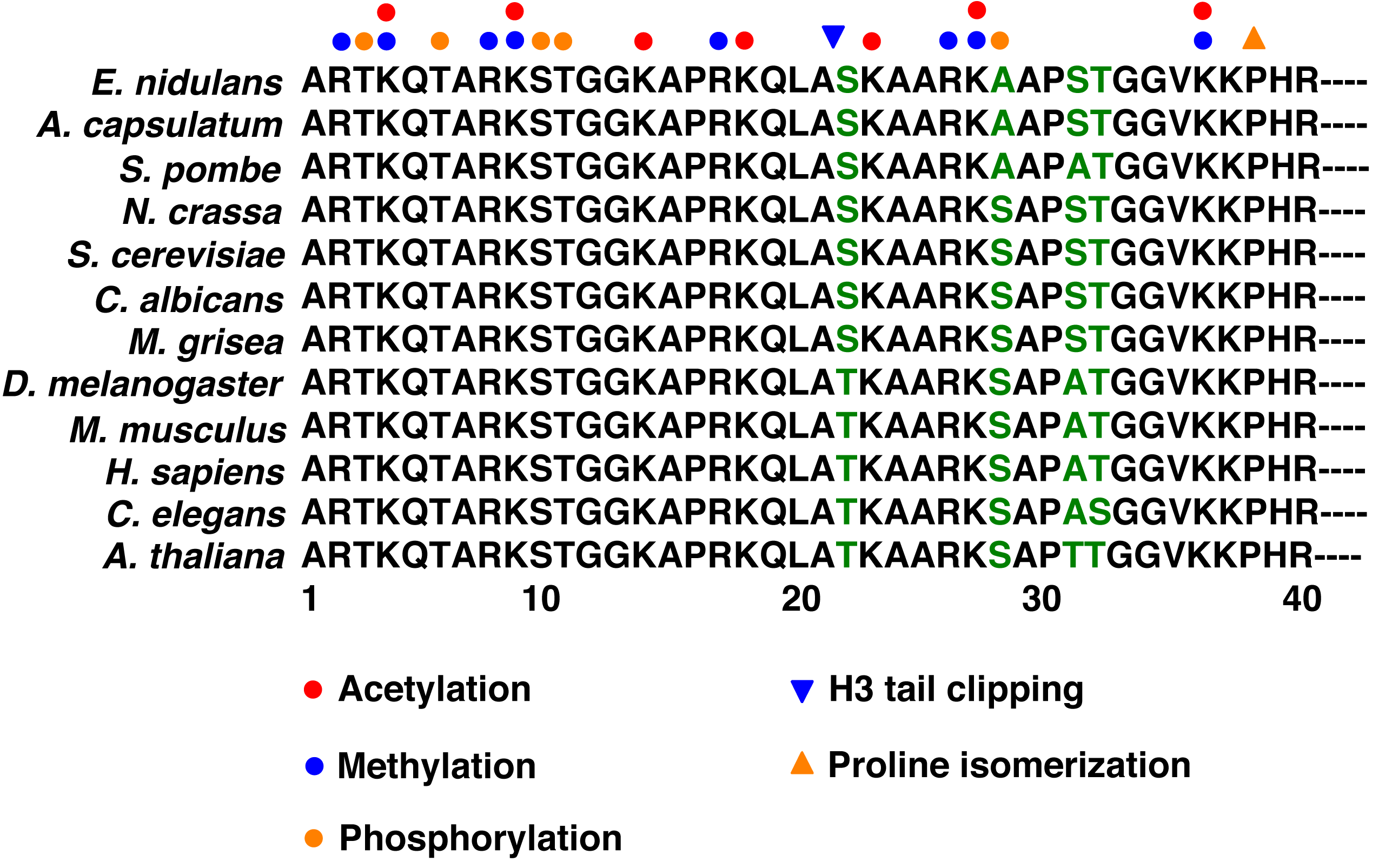

Supplement: Figure S1 — H3 amino-terminal tail residues are highly conserved. Alignment of H3 amino terminal tail from various eukaryotes including Emericella nidulans (gi 296337), Ajellomyces capsulatus (gi 9624455), Schizosacchromyces pombe (gi 5531473), Neurospora crassa (gi 18307450), Saccharomyces cerevisiae (gi 7019764), Candida albicans (gi 68490295), Magnaporthe grisea (gi 156630832), Drosophila melanogaster (gi 46397771), Mus musculus (gi 387198), Homo sapiens (gi 1568561), Caenorhabditis elegans (gi 12276045), Arabidopsis thaliana (gi 4490755). Variable residues are highlighted in green. Covalent modifications including methylation of K and R, acetylation of K and phosphorylation of S and T are highly conserved [10]. Recent studies have also reported proline isomerzation and clipping of H3 tail after A21 [80], [81]. (TIF) [file pgen.1002423.s001.tif]

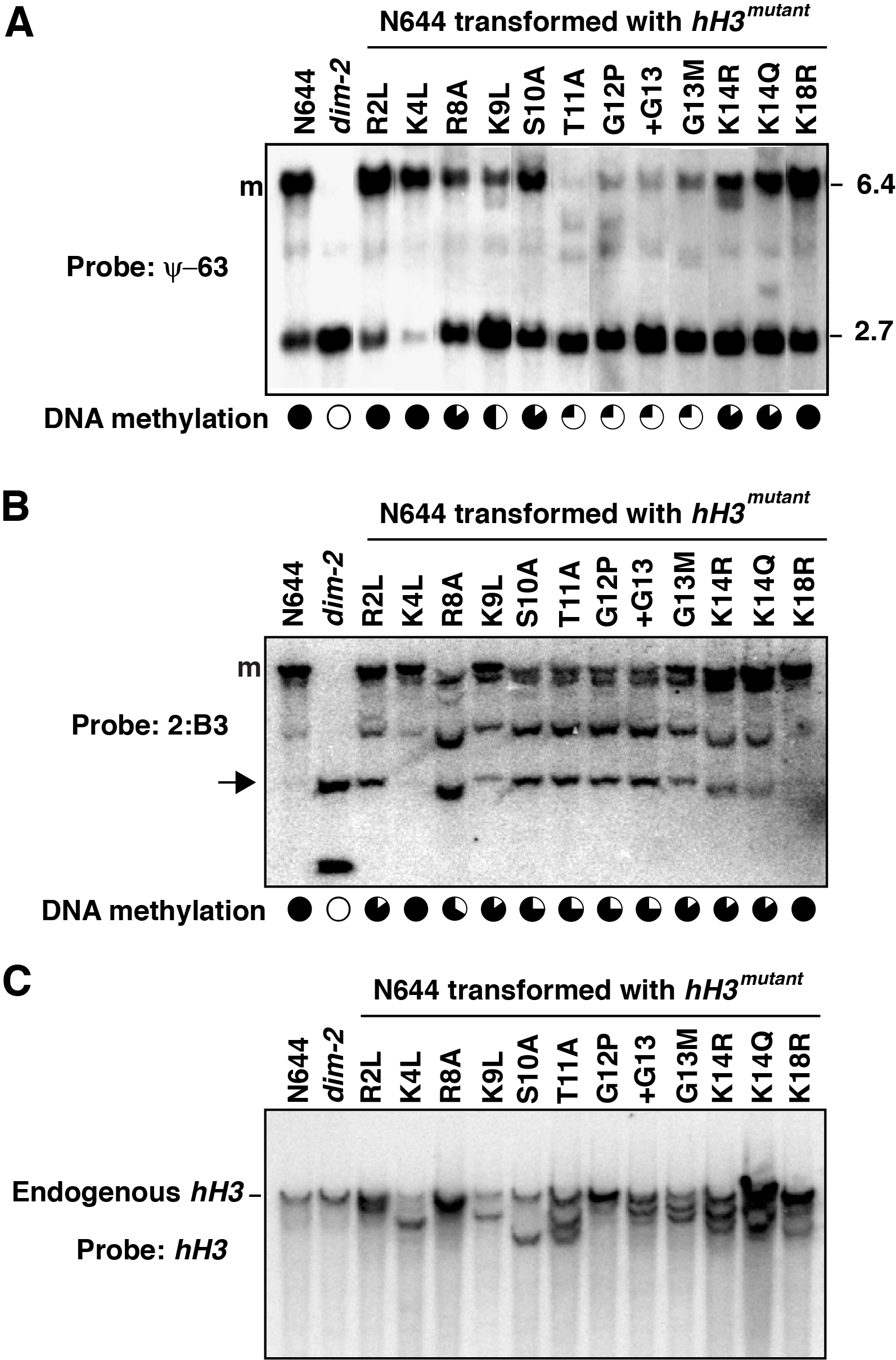

Supplement: Figure S2 — Southern analyses of hygromycin-resistant transformants containing mutant hH3 at an ectopic location and the wild-type gene at the native locus. Loss of DNA methylation in ψ63 (A) and 2:B3 (B) regions. DNA from selected hygromycin-resistant transformants was digested with methylation-sensitive restriction enzymes (BamHI and EcoRI for A; AvaII for B) and used for Southern hybridizations. The blots were probed for ψ63 [18]. Loss of DNA methylation was roughly quantified (pie graphs under autoradiograms) by measuring the ratio of the primary methylation (m) bands and the primary band representing unmethylated DNA (e.g. ratio of 6.4 and 2.7 kb bands for A). The strains are those described in Figure 1C. (C) Determination of mutant hH3 copies in selected transformants. DNA was digested with BamHI and EcoRI and used for Southern hybridizaitons probing with a fragment of the wild-type hH3 gene. (TIF) [file pgen.1002423.s002.tif]

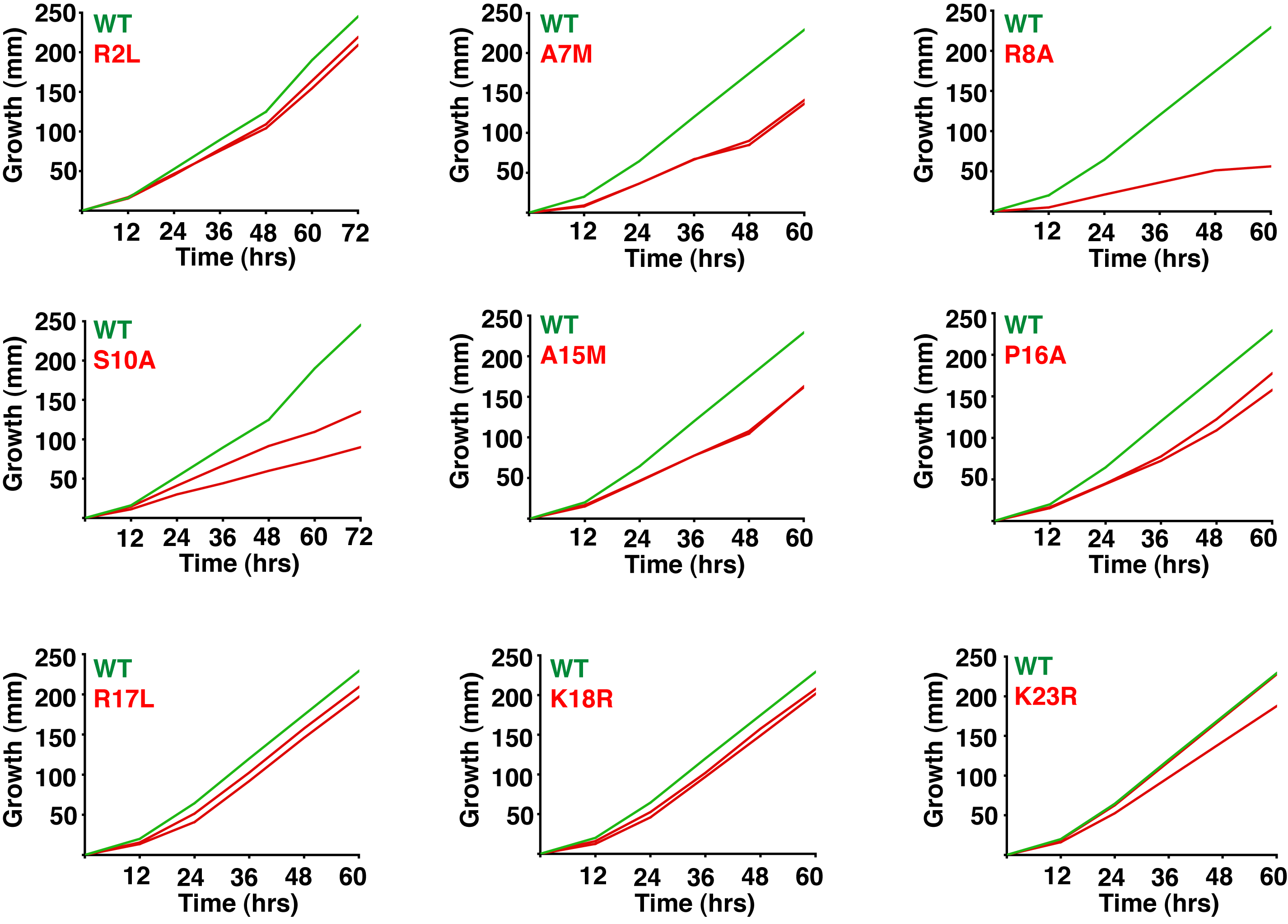

Supplement: Figure S3 — Poor growth of some H3 substitution strains. Wild type (N150) and strains with single copies of altered H3 genes [hH3R2L (N3517 and N3520), hH3A7M (N3530 and N3531), hH3R8A (N3542), hH3S10A (N3474 and N3481), hH3A15M (N3552 and N3553), hH3P16A (N3556 and N3557), hH3R19L (N3560 and N3561), hH3K18R (N3564 and N3565), hH3K23R (N3568 and N3569)] were grown on solid Vogel's N medium containing 1.5% sucrose in race tubes at 32°C [69]. Growth for each strain is an average of measurements in two tubes. (TIF) [file pgen.1002423.s003.tif]

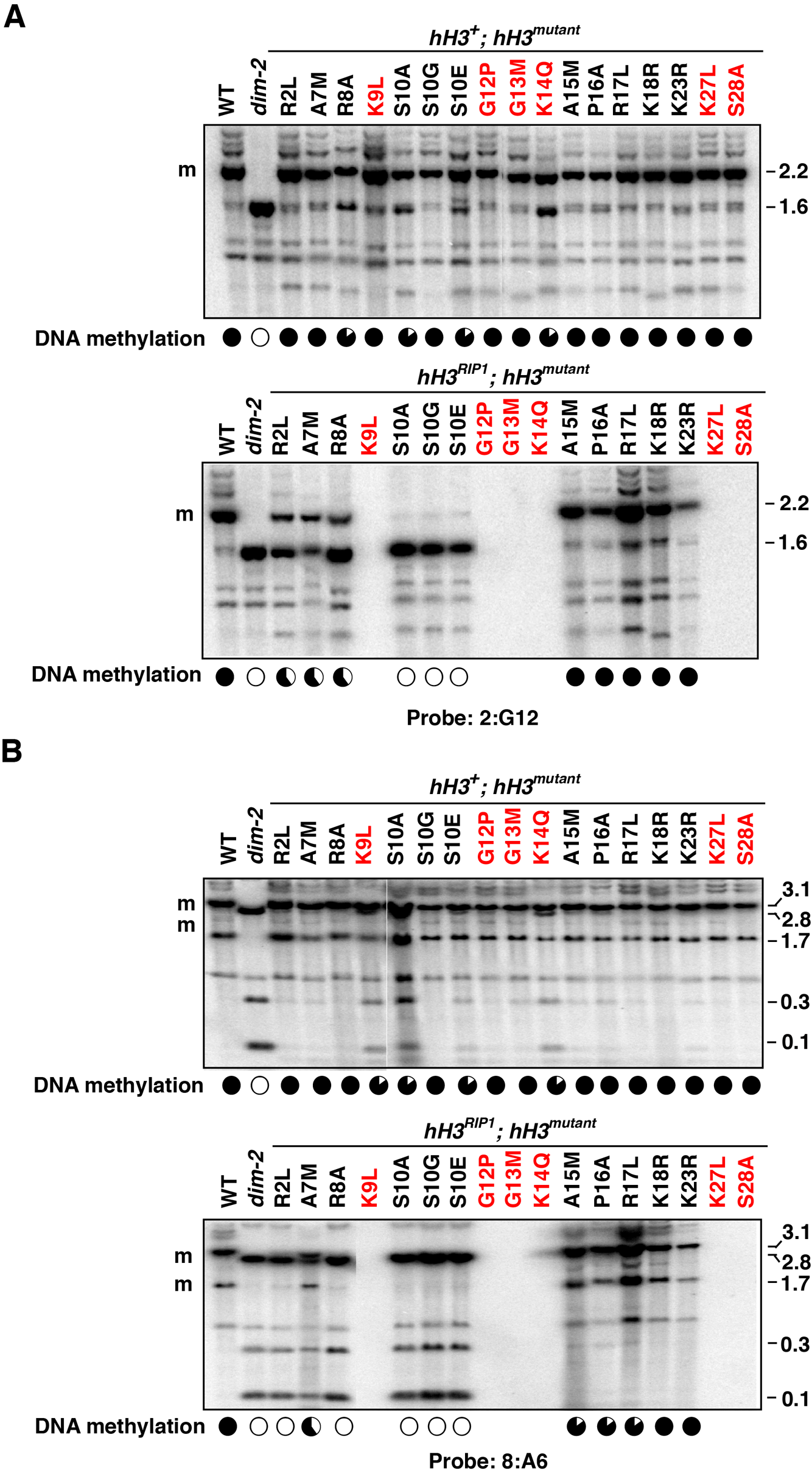

Supplement: Figure S4 — Loss of DNA methylation caused by H3 substitutions. DNA was digested with the 5mC-sensitive restriction enzyme AvaII (panel A) or BamHI and EcoRI (panel B) and used for Southern hybridizaitons probing with the indicated regions [18]. Strains are the same as listed in Figure 3C. Loss of DNA methylation was roughly quantified (pie graphs under autoradiograms) by measuring the ratio of the intensities of a methylation (m) band and the primary band representing unmethylated DNA (2.2 and 1.6 kb bands for 2:G12 region; 1.7 and 0.3 kb bands for the 8:A6 region). (TIF) [file pgen.1002423.s004.tif]

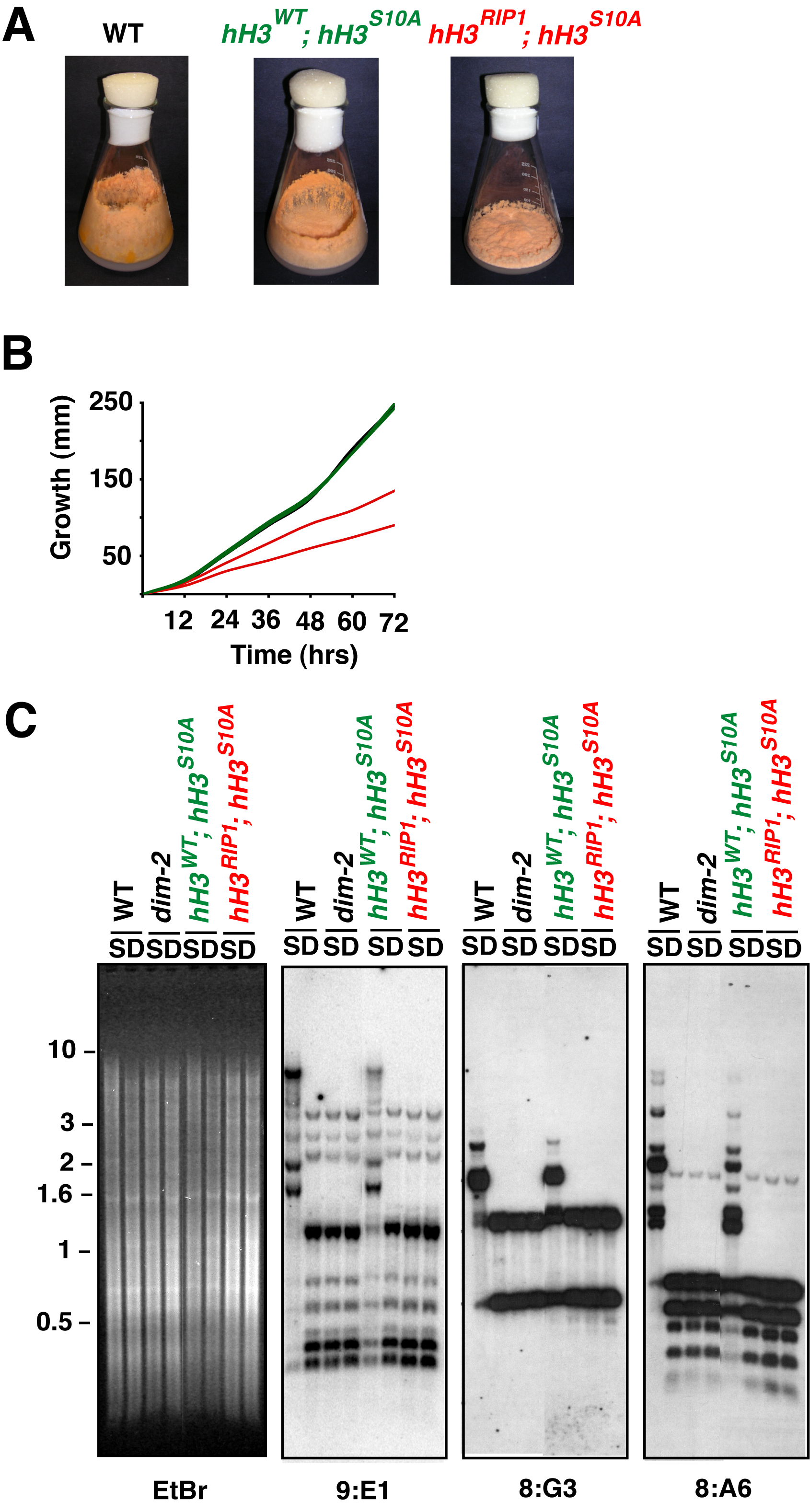

Supplement: Figure S5 — Comparison of a strain that contains just mutant H3 with a strain that contains a mixture of wild-type and mutant H3 proteins. A strain containing a mixture of wild-type and mutant H3 [hH3WT; hH3S10A (N3494), indicated in green] grows (panels A & B) and conidiates (panel A) better than a strain that contains just mutant H3 [hH3RIP1; hH3S10A (N3474); indicated in red]. All strains were grown on solid Vogel's N medium containing 1.5% sucrose for 7 days at 32°C. For the race tube measurements (B), results for wild-type (N150; black) and for two strains of each genotype [hH3RIP1; hH3S10A (N3474 and N3481) and hH3WT; hH3S10A (N3494 and N3495)] are shown. Growth for each strain is an average of measurements in two tubes. (C) Southern analysis of genomic DNA isolated from wild type (N150), dim-2 (N1860), a strain with both wild-type and mutant H3 [hH3WT; hH3S10A (N3494)], and a strain with only mutant H3 [hH3RIP1; hH3S10A (N3474)]. DNA was digested with the 5mC-sensitive restriction enzyme Sau3AI and 5mC-insensitive restriction enzyme DpnII and blots were probed for the methylated regions 8:A6, 8:G3 and 9:E1 [18]. (TIF) [file pgen.1002423.s005.tif]

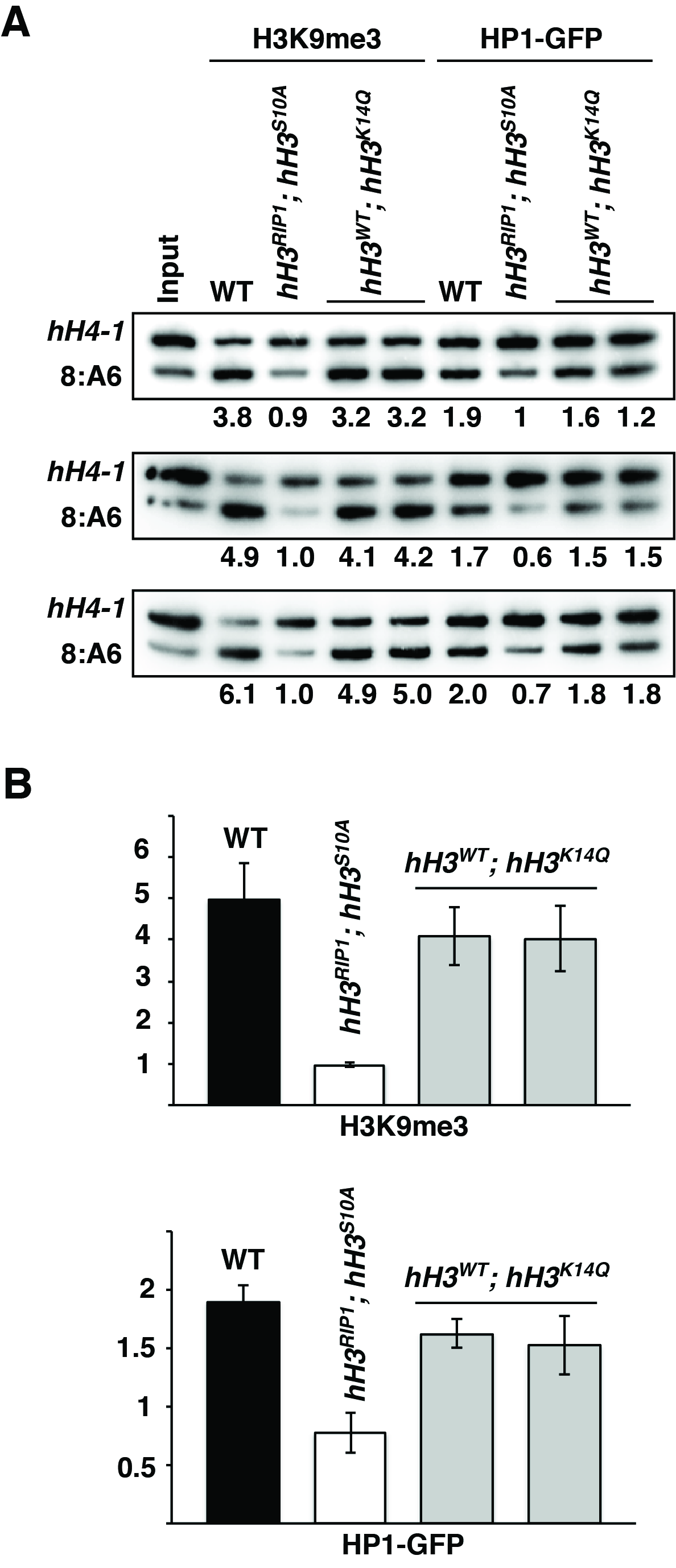

Supplement: Figure S6 — The heterozygous H3K14Q substitution mutant shows only slight reductions of H3K9me3 and HP1 in a genomic region that has reduced DNA methyation. (A) Chromatin IP (ChIP) was performed with wild-type (N150), hH3S10A (N3480) and hH3K14Q (N3550 and N3551) strains using antibodies to immunoprecipitate H3K9me3 or HP1-GFP. Three independent reactions on two biological replicas are shown. Primer pairs were used in duplex PCRs to amplify an unmethylated region (hH4-1) and a methylated region (8:A6) [15], [21]. The numbers indicate the relative enrichment of H3K9me3 or HP1-GFP at 8:A6 compared to hH4-1. (B) Quantitation of H3K9me3 levels and HP1 binding using data obtained from three independent duplex PCR reactions. (TIF) [file pgen.1002423.s006.tif]
