# Supplementary material for: Substitutions in the Amino-Terminal Tail of Neurospora Histone H3 Have Varied Effects on DNA Methylation
Source: PLoS Genet. 2011 Dec 29;7(12):e1002423. doi: 10.1371/journal.pgen.1002423 (PMC3248561; doi:10.1371/journal.pgen.1002423)
Supplement: Table S1 — Number of HygR colonies obtained in transformations with hH3 alleles. (DOCX) [file pgen.1002423.s007.docx]

**Table S1. Number of Hyg^R^ colonies obtained in transformations with *hH3* alleles.**

| **Substitution** | Transformation # | | | | | | | |
| --- | --- | --- | --- | --- | --- | --- | --- | --- |
|  | **1** | **2** | **3** | **4** | **5** | **6** | **7** | **8** |
| **R2L** | - | - | - | 3 | 12 | 69 | nd | Nd |
| **K4L** | nd | nd | - | - | - | - | - | - |
| **T6A** | nd | - | 2 | nd | - | - | nd | nd |
| **A7M** | - | - | Nd | - | - | nd | nd | nd |
| **R8A** | nd | - | 47 | - | 20 | 25 | nd | nd |
| **K9L** | 6 | 5 | - | - | - | - | - | 1 |
| **K9R** | - | - | - | - | 50 | - | 104 | 48 |
| **S10A** | 4 | - | 1 | - | 20 | 2 | - | 9 |
| **T11A** | 225 | - | 62 | 25 | 20 | 30 | nd | nd |
| **G12P** | - | - | 1 | - | 60 | 70 | nd | nd |
| **+G13** | - | - | 13 | nd | - | 30 | 4 | 78 |
| **G13M** | 2 | 3 | 4 | - | 50 | 100 | 21 | 98 |
| **K14Q** | 3 | 8 | 21 | - | 14 | 30 | 90 | 100 |
| **K14R** | 160 | - | nd | 15 | 150 | 180 | nd | nd |
| **A15M** | - | - | - | - | - | 5 | nd | nd |
| **P16A** | nd | nd | - | 1 | - | - | nd | nd |
| **R17L** | nd | - | - | 2 | - | nd | nd | nd |
| **K18Q** | nd | nd | 1 | - | - | - | - | 2 |
| **K18R** | nd | nd | 28 | - | - | - | - | - |
| **K23Q** | nd | nd | - | - | - | - | - | - |
| **K23R** | nd | nd | 15 | - | - | - | - | - |
| **K27L** | nd | nd | - | - | - | - | - | - |
| **S28A** | nd | nd | - | - | - | - | - | - |
| **K36A** | nd | nd | - | - | - | - | - | - |

nd = not done

- = no colonies
