# Supplementary material for: Substitutions in the Amino-Terminal Tail of Neurospora Histone H3 Have Varied Effects on DNA Methylation
Source: PLoS Genet. 2011 Dec 29;7(12):e1002423. doi: 10.1371/journal.pgen.1002423 (PMC3248561; doi:10.1371/journal.pgen.1002423)
Supplement: Table S2 — Strains used in this study. (DOCX) [file pgen.1002423.s008.docx]

**Table S2. Strains used in this study**

| **Strain** | **Genotype** | Source |
| --- | --- | --- |
| **N39** | *fl ; mat A* | Our lab  collection |
| **N40** | *fl ; mat a* |  |
| **N150** | (74-OR23-IV) *mat A* |  |
| **N625** | *his-3 mat a* |  |
| **N644** | *am^132^ inl; [(am/hph/am)^ec42pJI2^]^RIP^ ; mat A* |  |
| **N2240** | *rid^RIP4^ his-3 mat A* |  |
| **N2257** | *rid^RIP4^ his-3 mat a* |  |
| **N2264** | *his-3; dim-5 leu-2 pan-1 ; mat a* |  |
| **N2556** | *rid^RIP4^ his-3^+^::hpo^+^-sgfp^+^; mat A* |  |
| **N2879** | *pan-2::hph^+^-tk^+^ ; mat A* |  |
| **N3016** | *Sad-1 his-3 mat a* |  |
| **N3079** | *am^132^ inl; [(am/hph/am)^ec42pJI2^]^RIP^(hH3^R2L^)^ec^ ; mat A* | This study |
| **N3080** | *am^132^ inl; [(am/hph/am)^ec42pJI2^]^RIP^(hH3^R2L^)^ec^ ; mat A* | This study |
| **N3081** | *am^132^ inl; [(am/hph/am)^ec42pJI2^]^RIP^(hH3^K4L^)^ec^ ; mat A* | This study |
| **N3082** | *am^132^ inl; [(am/hph/am)^ec42pJI2^]^RIP^(hH3^R8A^)^ec^ ; mat A* | This study |
| **N3083** | *am^132^ inl; [(am/hph/am)^ec42pJI2^]^RIP^(hH3^K9L^)^ec^ ; mat A* | This study |
| **N3084** | *am^132^ inl; [(am/hph/am)^ec42pJI2^]^RIP^(hH3^K9L^)^ec^ ; mat A* | This study |
| **N3085** | *am^132^ inl; [(am/hph/am)^ec42pJI2^]^RIP^(hH3^S10A^)^ec^ ; mat A* | This study |
| **N3086** | *am^132^ inl; [(am/hph/am)^ec42pJI2^]^RIP^(hH3^T11A^)^ec^ ; mat A* | This study |
| **N3087** | *am^132^ inl; [(am/hph/am)^ec42pJI2^]^RIP^(hH3^T11A^)^ec^ ; mat A* | This study |
| **N3088** | *am^132^ inl; [(am/hph/am)^ec42pJI2^]^RIP^(hH3^G12P^)^ec^ ; mat A* | This study |
| **N3089** | *am^132^ inl; [(am/hph/am)^ec42pJI2^]^RIP^(hH3^G12P^)^ec^ ; mat A* | This study |
| **N3090** | *am^132^ inl; [(am/hph/am)^ec42pJI2^]^RIP^(hH3^+G13^)^ec^ ; mat A* | This study |
| **N3091** | *am^132^ inl; [(am/hph/am)^ec42pJI2^]^RIP^(hH3^G13M^)^ec^ ; mat A* | This study |
| **N3092** | *am^132^ inl; [(am/hph/am)^ec42pJI2^]^RIP^(hH3^G13M^)^ec^ ; mat A* | This study |
| **N3093** | *am^132^ inl; [(am/hph/am)^ec42pJI2^]^RIP^(hH3^K14R^)^ec^ ; mat A* | This study |
| **N3094** | *am^132^ inl; [(am/hph/am)^ec42pJI2^]^RIP^(hH3^K14R^)^ec^ ; mat A* | This study |
| **N3095** | *am^132^ inl; [(am/hph/am)^ec42pJI2^]^RIP^(hH3^K14Q^)^ec^ ; mat A* | This study |
| **N3096** | *am^132^ inl; [(am/hph/am)^ec42pJI2^]^RIP^(hH3^K14Q^)^ec^ ; mat A* | This study |
| **N3097** | *am^132^ inl; [(am/hph/am)^ec42pJI2^]^RIP^(hH3^K18R^)^ec^ ; mat A* | This study |
| **N3098** | *am^132^ inl; [(am/hph/am)^ec42pJI2^]^RIP^(hH3^K18R^)^ec^ ; mat A* | This study |
| **N3099** | *am^132^ inl; [(am/hph/am)^ec42pJI2^]^RIP^(hH3^K23R^)^ec^ ; mat A* | This study |
| **N3100** | *am^132^ inl; [(am/hph/am)^ec42pJI2^]^RIP^(hH3^K23R^)^ec^ ; mat A* | This study |
| **N3474** | *Sad-1 his-3****^+^****::hH3^S10A^* *; hH3^RIP1^ mat a* | Our lab  collection |
| **N3475** | *rid^RIP4^ his-3****^+^****::hH3^S10G^* *mat A* |  |
| **N3476** | *rid^RIP4^ his-3****^+^****::hH3^S10E^* *mat A* |  |
| **N3477** | *rid^RIP4^ his-3****^+^****::hH3^S10G^* *; hH3^RIP1^  mat A* |  |
| **N3478** | *rid^RIP4^ his-3****^+^****::hH3^S10E^* *; hH3^RIP1^ ; mat A* |  |
| **N3480** | *his-3; pan-2^+^::hpo^+^-sgfp^+^ ; mat A* |  |
| **N3481** | *Sad-1 his-3^+^::hH3^S10A^ ; hH3^RIP1^ ; pan-2^+^::hpo^+^-sgfp^+^ ; mat a* |  |
| **N3492** | *rid^RIP4^ his-3****^+^****::hH3^WT^* ***;*** *mat A* | This study |
| **N3493** | *rid^RIP4^ his-3****^+^****::hH3^WT^* ***;*** *mat A* | This study |
| **N3494** | *Sad-1 his-3****^+^****::hH3^S10A^* *mat a* | This study |
| **N3495** | *Sad-1 his-3****^+^****::hH3^S10A^* *mat a* | This study |
| **N3496** | *rid^RIP4^ his-3****^+^****::hH3^S10G^* *; hH3^RIP1^ ; mat A* | This study |
| **N3497** | *Sad-1 his-3****^+^****::hH3^S10G^*  *mat a* | This study |
| **N3498** | *Sad-1 his-3****^+^****::hH3^S10G^* *mat a* | This study |
| **N3499** | *rid^RIP4^ his-3****^+^****::hH3^S10E^* *; hH3^RIP1^ ; mat A* | This study |
| **N3500** | *Sad-1 his-3****^+^****::hH3^S10E^* ***;*** *mat a* | This study |
| **N3501** | *Sad-1 his-3****^+^****::hH3^S10E^* ***;*** *mat a* | This study |
| **N3502** | *rid^RIP4^ his-3****^+^****::hH3^R2L^* *; pan-2^+^::hpo^+^-sgfp^+^ ; mat A* | This study |
| **N3503** | *rid^RIP4^ his-3****^+^****::hH3^T6A^* *; pan-2^+^::hpo^+^-sgfp^+^ ; mat A* | This study |
| **N3504** | *rid^RIP4^ his-3****^+^****::hH3^A7M^* *; pan-2^+^::hpo^+^-sgfp^+^ ; mat A* | This study |
| **N3505** | *rid^RIP4^ his-3****^+^****::hH3^R8A^ ; pan-2^+^::hpo^+^-sgfp^+^ ; mat A* | This study |
| **N3506** | *rid^RIP4^ his-3****^+^****::hH3^K9L^* *; pan-2^+^::hpo^+^-sgfp^+^ ; mat A* | This study |
| **N3507** | *rid^RIP4^ his-3****^+^****::hH3^G12P^* *; pan-2^+^::hpo^+^-sgfp^+^ ; mat A* | This study |
| **N3508** | *rid^RIP4^ his-3****^+^****::hH3^G13M^* *; pan-2^+^::hpo^+^-sgfp^+^ ; mat A* | This study |
| **N3509** | *rid^RIP4^ his-3****^+^****::hH3^K14Q^* *; pan-2^+^::hpo^+^-sgfp^+^ ; mat A* | This study |
| **N3510** | *rid^RIP4^ his-3****^+^****::hH3^A15M^* *; pan-2^+^::hpo^+^-sgfp^+^ ; mat A* | This study |
| **N3511** | *rid^RIP4^ his-3****^+^****::hH3^P16A^ ; pan-2^+^::hpo^+^-sgfp^+^ ; mat A* | This study |
| **N3512** | *rid^RIP4^ his-3****^+^****::hH3^R17L^* *; pan-2^+^::hpo^+^-sgfp^+^ ; mat A* | This study |
| **N3513** | *rid^RIP4^ his-3****^+^****::hH3^K18R^ ; pan-2^+^::hpo^+^-sgfp^+^ ; mat A* | This study |
| **N3514** | *rid^RIP4^ his-3****^+^****::hH3^K23R^ ; pan-2^+^::hpo^+^-sgfp^+^ ; mat A* | This study |
| **N3515** | *rid^RIP4^ his-3****^+^****::hH3^K237L^ ; pan-2^+^::hpo^+^-sgfp^+^ ; mat A* | This study |
| **N3516** | *rid^RIP4^ his-3****^+^****::hH3^S28A^ ; pan-2^+^::hpo^+^-sgfp^+^ ; mat A* | This study |
| **N3517** | *rid^RIP4^ his-3****^+^****::hH3^R2L^* *; hH3^RIP1^ ; pan-2^+^::hpo^+^-sgfp^+^; mat A* | This study |
| **N3518** | *rid^RIP4^ his-3****^+^****::hH3^R2L^* *; hH3^RIP1^ ; pan-2^+^::hpo^+^-sgfp^+^; mat A* | This study |
| **N3519** | *rid^RIP4^ his-3****^+^****::hH3^R2L^* *; hH3^RIP1^ ; pan-2^+^::hpo^+^-sgfp^+^; mat A* | This study |
| **N3520** | *rid^RIP4^ his-3****^+^****::hH3^R2L^* *; hH3^RIP1^ ; pan-2^+^::hpo^+^-sgfp^+^; mat A* | This study |
| **N3521** | *rid^RIP4^ his-3****^+^****::hH3^R2L^* *; hH3^RIP1^ ; pan-2^+^::hpo^+^-sgfp^+^; mat A* | This study |
| **N3522** | *rid^RIP4^ his-3****^+^****::hH3^R2L^* *; hH3^RIP1^ ; pan-2^+^::hpo^+^-sgfp^+^; mat A* | This study |
| **N3523** | *rid^RIP4^ his-3****^+^****::hH3^R2L^* *; hH3^RIP1^ ; pan-2^+^::hpo^+^-sgfp^+^; mat A* | This study |
| **N3524** | *rid^RIP4^ his-3****^+^****::hH3^R2L^* *; pan-2^+^::hpo^+^-sgfp^+^; mat A* | This study |
| **N3525** | *rid^RIP4^ his-3****^+^****::hH3^R2L^* *; pan-2^+^::hpo^+^-sgfp^+^; mat A* | This study |
| **N3526** | *rid^RIP4^ his-3****^+^****::hH3^T6A^* *; hH3^RIP1^ ; pan-2^+^::hpo^+^-sgfp^+^; mat A* | This study |
| **N3527** | *rid^RIP4^ his-3****^+^****::hH3^T6A^* *; hH3^RIP1^ ; pan-2^+^::hpo^+^-sgfp^+^; mat A* | This study |
| **N3528** | *rid^RIP4^ his-3****^+^****::hH3^T6A^* *; pan-2^+^::hpo^+^-sgfp^+^; mat A* | This study |
| **N3529** | *rid^RIP4^ his-3****^+^****::hH3^T6A^* *; pan-2^+^::hpo^+^-sgfp^+^; mat A* | This study |
| **N3530** | *rid^RIP4^ his-3****^+^****::hH3^A7M^* *; hH3^RIP1^ ; pan-2^+^::hpo^+^-sgfp^+^; mat A* | This study |
| **N3531** | *rid^RIP4^ his-3****^+^****::hH3^A7M^* *; hH3^RIP1^ ; pan-2^+^::hpo^+^-sgfp^+^; mat A* | This study |
| **N3532** | *rid^RIP4^ his-3****^+^****::hH3^A7M^* *; hH3^RIP1^ ; pan-2^+^::hpo^+^-sgfp^+^; mat A* | This study |
| **N3533** | *rid^RIP4^ his-3****^+^****::hH3^A7M^* *; hH3^RIP1^ ; pan-2^+^::hpo^+^-sgfp^+^; mat A* | This study |
| **N3534** | *rid^RIP4^ his-3****^+^****::hH3^A7M^* *; hH3^RIP1^ ; pan-2^+^::hpo^+^-sgfp^+^; mat A* | This study |
| **N3535** | *rid^RIP4^ his-3****^+^****::hH3^A7M^* *; hH3^RIP1^ ; pan-2^+^::hpo^+^-sgfp^+^; mat A* | This study |
| **N3536** | *rid^RIP4^ his-3****^+^****::hH3^A7M^* *; hH3^RIP1^ ; pan-2^+^::hpo^+^-sgfp^+^; mat A* | This study |
| **N3537** | *rid^RIP4^ his-3****^+^****::hH3^A7M^* *; hH3^RIP1^ ; pan-2^+^::hpo^+^-sgfp^+^; mat A* | This study |
| **N3538** | *rid^RIP4^ his-3****^+^****::hH3^A7M^* *; hH3^RIP1^ ; pan-2^+^::hpo^+^-sgfp^+^; mat A* | This study |
| **N3539** | *rid^RIP4^ his-3****^+^****::hH3^A7M^* *; hH3^RIP1^ ; pan-2^+^::hpo^+^-sgfp^+^; mat A* | This study |
| **N3540** | *rid^RIP4^ his-3****^+^****::hH3^A7M^* *; pan-2^+^::hpo^+^-sgfp^+^; mat A* | This study |
| **N3541** | *rid^RIP4^ his-3****^+^****::hH3^A7M^* *; pan-2^+^::hpo^+^-sgfp^+^; mat A* | This study |
| **N3542** | *rid^RIP4^ his-3****^+^****::hH3^R8A^* *; hH3^RIP1^ ; pan-2^+^::hpo^+^-sgfp^+^; mat A* | This study |
| **N3543** | *rid^RIP4^ his-3****^+^****::hH3^R8A^ ; pan-2^+^::hpo^+^-sgfp^+^; mat A* | This study |
| **N3544** | *rid^RIP4^ his-3****^+^****::hH3^K9L^* *; pan-2^+^::hpo^+^-sgfp^+^; mat A* | This study |
| **N3545** | *rid^RIP4^ his-3****^+^****::hH3^K9L^* *; pan-2^+^::hpo^+^-sgfp^+^; mat A* | This study |
| **N3546** | *rid^RIP4^ his-3****^+^****::hH3^G12P^* *; pan-2^+^::hpo^+^-sgfp^+^; mat A* | This study |
| **N3547** | *rid^RIP4^ his-3****^+^****::hH3^G12P^* *; pan-2^+^::hpo^+^-sgfp^+^; mat A* | This study |
| **N3548** | *rid^RIP4^ his-3****^+^****::hH3^G13M^* *; pan-2^+^::hpo^+^-sgfp^+^; mat A* | This study |
| **N3549** | *rid^RIP4^ his-3****^+^****::hH3^G13M^* *; pan-2^+^::hpo^+^-sgfp^+^; mat A* | This study |
| **N3550** | *rid^RIP4^ his-3****^+^****::hH3^K14Q^* *; pan-2^+^::hpo^+^-sgfp^+^; mat A* | This study |
| **N3551** | *rid^RIP4^ his-3****^+^****::hH3^K14Q^* *; pan-2^+^::hpo^+^-sgfp^+^; mat A* | This study |
| **N3552** | *rid^RIP4^ his-3****^+^****::hH3^A15M^* *; hH3^RIP1^ ; pan-2^+^::hpo^+^-sgfp^+^; mat A* | This study |
| **N3553** | *rid^RIP4^ his-3****^+^****::hH3^A15M^* *; hH3^RIP1^ ; pan-2^+^::hpo^+^-sgfp^+^; mat A* | This study |
| **N3554** | *rid^RIP4^ his-3****^+^****::hH3^A15M^* *; pan-2^+^::hpo^+^-sgfp^+^; mat A* | This study |
| **N3555** | *rid^RIP4^ his-3****^+^****::hH3^A15M^* *; pan-2^+^::hpo^+^-sgfp^+^; mat A* | This study |
| **N3556** | *rid^RIP4^ his-3****^+^****::hH3^P16A^* *; hH3^RIP1^ ; pan-2^+^::hpo^+^-sgfp^+^; mat A* | This study |
| **N3557** | *rid^RIP4^ his-3****^+^****::hH3^P16A^* *; hH3^RIP1^ ; pan-2^+^::hpo^+^-sgfp^+^; mat A* | This study |
| **N3558** | *rid^RIP4^ his-3****^+^****::hH3^P16A^ ; pan-2^+^::hpo^+^-sgfp^+^; mat A* | This study |
| **N3559** | *rid^RIP4^ his-3****^+^****::hH3^P16A^ ; pan-2^+^::hpo^+^-sgfp^+^; mat A* | This study |
| **N3560** | *rid^RIP4^ his-3****^+^****::hH3^R17L^* *; hH3^RIP1^ ; pan-2^+^::hpo^+^-sgfp^+^; mat A* | This study |
| **N3561** | *rid^RIP4^ his-3****^+^****::hH3^R17L^* *; hH3^RIP1^ ; pan-2^+^::hpo^+^-sgfp^+^; mat A* | This study |
| **N3562** | *rid^RIP4^ his-3****^+^****::hH3^R17L^* *; pan-2^+^::hpo^+^-sgfp^+^; mat A* | This study |
| **N3563** | *rid^RIP4^ his-3****^+^****::hH3^R17L^* *; pan-2^+^::hpo^+^-sgfp^+^; mat A* | This study |
| **N3564** | *rid^RIP4^ his-3****^+^****::hH3^K18R^* *; hH3^RIP1^ ; pan-2^+^::hpo^+^-sgfp^+^; mat A* | This study |
| **N3565** | *rid^RIP4^ his-3****^+^****::hH3^K18R^* *; hH3^RIP1^ ; pan-2^+^::hpo^+^-sgfp^+^; mat A* | This study |
| **N3566** | *rid^RIP4^ his-3****^+^****::hH3^K18R^ ; pan-2^+^::hpo^+^-sgfp^+^; mat A* | This study |
| **N3567** | *rid^RIP4^ his-3****^+^****::hH3^K18R^ ; pan-2^+^::hpo^+^-sgfp^+^; mat A* | This study |
| **N3568** | *rid^RIP4^ his-3****^+^****::hH3^K23R^* *; hH3^RIP1^ ; pan-2^+^::hpo^+^-sgfp^+^; mat A* | This study |
| **N3569** | *rid^RIP4^ his-3****^+^****::hH3^K23R^* *; hH3^RIP1^ ; pan-2^+^::hpo^+^-sgfp^+^; mat A* | This study |
| **N3570** | *rid^RIP4^ his-3****^+^****::hH3^K23R^ ; pan-2^+^::hpo^+^-sgfp^+^; mat A* | This study |
| **N3571** | *rid^RIP4^ his-3****^+^****::hH3^K23R^ ; pan-2^+^::hpo^+^-sgfp^+^; mat A* | This study |
| **N3572** | *rid^RIP4^ his-3****^+^****::hH3^K237L^; pan-2^+^::hpo^+^-sgfp^+^; mat A* | This study |
| **N3573** | *rid^RIP4^ his-3****^+^****::hH3^S28A^ ; pan-2^+^::hpo^+^-sgfp^+^; mat A* | This study |
| **N3574** | *rid^RIP4^ his-3****^+^****::hH3^S28A^ ; pan-2^+^::hpo^+^-sgfp^+^; mat A* | This study |
|  |  |  |
