# Supplementary material for: Substitutions in the Amino-Terminal Tail of Neurospora Histone H3 Have Varied Effects on DNA Methylation
Source: PLoS Genet. 2011 Dec 29;7(12):e1002423. doi: 10.1371/journal.pgen.1002423 (PMC3248561; doi:10.1371/journal.pgen.1002423)
Supplement: Table S3 — Primers used in this study. (DOCX) [file pgen.1002423.s009.docx]

**Table S3. Primers used in this study**

| **Primer** | **Description** | **Sequence** |
| --- | --- | --- |
| 1068 | H3A10-REV | CTTGCCACCGGTGGCCTTGCGGGCGGT |
| 1069 | H3R14-REV | CTTACGGGGGGCACGGCCACCGGTGGA |
| 1070 | H3Q14-FWD | TCCACCGGTGGCCAGGCCCCCCGTAAG |
| 1070 | H3Q14-FWD | TCCACCGGTGGCCAGGCCCCCCGTAAG |
| 1071 | H3A10-FWD | ACCGCCCGCAAGGCCACCGGTGGCAAG |
| 1072 | H3R14-FWD | TCCACCGGTGGCCGTGCCCCCCGTAAG |
| 1073 | H3Q14-REV | CTTACGGGGGGCCTGGCCACCGGTGGA |
| 1074 | H3tail-HIS-FWD | CATGCCATGGCCCGCACTAAGCAGACC |
| 1074 | H3tail-HIS-FWD | CATGCCATGGCCCGCACTAAGCAGACC |
| 1075 | H3tail-HIS-REV | CGGGATCCACGGTGGGGCTTCTTGACACCG |
| 1076 | H3+G12-13-FWD | CGCAAGTCCACCGGTGGTGGCAAGGCCC |
| 1076 | H3+G12-13-FWD | CGCAAGTCCACCGGTGGTGGCAAGGCCC |
| 1077 | H3+G12-13-REV | GGGCCTTGCCACCACCGGTGGACTTGCG |
| 1079 | H3L4-FWD | ATGGCCCGCACTCTCCAGACCGCCCGC |
| 1079 | H3L4-FWD | ATGGCCCGCACTCTCCAGACCGCCCGC |
| 1080 | H3L4-REV | GCGGGCGGTCTGGAGAGTGCGGGCCAT |
| 1081 | H3L27-FWD | GCTTCCAAGGCTGCCCGCCTCTCCGCCC |
| 1082 | H3L27-REV | GGGCGGAGAGGCGGGCAGCCTTGGAAGC |
| 1083 | H3Q18-FWD | CCCGTCAGCAGCTCGCTTCCAAGGCTG |
| 1084 | H3Q18-REV | CAGCCTTGGAAGCGAGCTGCTGACGGG |
| 1085 | H3R14-FWD | CCCGTCGTCAGCTCGCTTCCAAGGCTG |
| 1086 | H3R14-REV | CAGCCTTGGAAGCGAGCTGACGACGGG |
| 1087 | H3Q23-FWD | CAGCTCGCTTCCCAGGCTGCCCGCAAG |
| 1088 | H3Q23-REV | CTTGCGGGCAGCCTGGGAAGCGAGCTG |
| 1089 | H3R23-FWD | CAGCTCGCTTCCCGTGCTGCCCGCAAG |
| 1090 | H3R23-REV | CTTGCGGGCAGCACGGGAAGCGAGCTG |
| 1091 | H3A28-FWD | GCTGCCCGCAAGGCCGCCCCCTCCACC |
| 1091 | H3A28-FWD | GCTGCCCGCAAGGCCGCCCCCTCCACC |
| 1092 | H3A28-REV | GGTGGAGGGGGCCTTGCGGGCAGC |
| 1093 | H3L36-FWD | ACCGGCGGTGTCCTCAAGCCCCACCGT |
| 1094 | H3L36-REV | ACGGTGGGGCTTGAGGACACCGCCGGT |
| 1127 | H3-ORF-5' | AAACACGTTATCAACACACATAAACC |
| 1128 | H3-ORF-3' | CTGGAAGGGGAGCTTGCGGA |
| 1129 | H3R9-FWD | GCCACCGGTGGAACGGCGGGCGGTCTG |
| 1130 | H3R9-REV | CAGACCGCCCGCCGTTCCACCGGTGGC |
| 1131 | H3L9-FWD | GCCACCGGTGGAGAGGCGGGCGGTCTG |
| 1132 | H3L9-REV | CAGACCGCCCGCCTCTCCACCGGTGGC |
| 1173 | H32L-FWD | CCATCACAATGGCCCTCACTAAGCAGACCGC |
| 1174 | H32L-REV | GCGGTCTGCTTAGTGAGGGCCATTGTGATGG |
| 1175 | H36A-FWD | CGCACTAAGCAGGCCGCCCGCAAGTCC |
| 1176 | H36A-REV | GGACTTGCGGGCGGCCTGCTTAGTGCG |
| 1177 | H37M-FWD | GCACTAAGCAGACCATGAGCAAGTCCACCGG |
| 1178 | H37M-REV | CCGGTGGACTTGCGCATGGTCTGCTTAGTGC |
| 1179 | H38A-FWD | AAGCAGACCGCCGCCAAGTCCACCGGT |
| 1180 | H38A-REV | ACCGGTGGACTTGGCGGCGGTCTGCTT |
| 1181 | H311A-FWD | GCCCGCAAGTCCGCCGGTGGCAAGGCC |
| 1182 | H311A-REV | GGCCTTGCCACCGGCGGACTTGCGGGC |
| 1183 | H312P-FWD | CGCAAGTCCACCCCCGGCAAGGCCCCC |
| 1184 | H312P-REV | GGGGCCCTTGCCGGGGGTGGACTTGCG |
| 1185 | H313M-FWD | AAGTCCACCGGTATGAAGGCCCCCCGT |
| 1186 | H313M-REV | ACGGGGGGCCTTCATACCGGTGGACTT |
| 1187 | H315M-FWD | ACCGGTGGCAAGATGCCCCGTAAGCAG |
| 1188 | H315M-REV | CTGCTTACGGGGCATCTTGCCACCGGT |
| 1189 | H316A-FWD | GGTGGCAAGGCCGCCCGTAAGCAGCTC |
| 1190 | H316A-REV | GAGCTGCTTACGGGCGGCCTTGCCACC |
| 1191 | H317L-FWD | GGCAAGGCCCCCCTCAAGCAGCTCGCT |
| 1192 | H317L-REV | AGCGAGCTGCTTGAGGGGGGCCTTGCC |
| 1219 | H3(1-57)EX-FWD | CGGGATCCCATATGGCCCGCACTAAGCAGACCGCC |
| 1220 | H3(1-57)EX-REV | CGGAATTCTTAGGACTTCTGGTAGCGACGAATCTC |
| 1221 | H316A-REV | GAG CTG CTT ACG GGC GGC CTT GCC ACC |
| 1488 | H3flEcoR | gccgaattcgaacaaggtccgtctgtctc |
| 1490 | H3flBspFmtr | caagtgagttcttccggatcacgcgacgtgactttg |
| 1492 | H3flNheFhis | accccatagccgtgcacggctagctcacgcgacgtgactttg |
| 1496 | H3flNheFinl | ccgtgatacaaactcctagctagctcacgcgacgtgactttg |
| 1809 | H3AF | cccaataagttcaaaccg |
| 1810 | H3AR | tccagcccaattcctccg |
| 1811 | H3BF | gggagttgcaaatcaacc |
| 1812 | H3BR | ctgcttagtgcgggccat |
| 1813 | H3CF | atggcccgcactaagcag |
| 1814 | H3CR | tcctgggcaatctcacgg |
| 2105 | H3AF | CCCAATAAGTTCAAACCG |
| 2106 | H3AR | TCCAGCCCAATTCCTCCG |
| 2107 | H3BF | GGGAGTTGCAAATCAACC |
| 2108 | H3BR | CTGCTTAGTGCGGGCCAT |
| 2109 | H3CF | ATGGCCCGCACT AAGCAG |
| 2110 | H3CR | TCCTGGGCAATCTCACGG |
| 2111 | H3DF | CCGTGAGATTGCCCAGGA |
| 2112 | H3DR | GTGATATAACCCCAGAAT |
| 2189 | H3S10GFor | ACCGCCCGCAAGGGCACCGGTGGCAAG |
| 2190 | H3S10GRev | CTTGCCACCGGTGCCCTTGCGGGCGGT |
| 2191 | H3S10EFor | ACCGCCCGCAAGGAGACCGGTGGCAAG |
| 2192 | H3S10ERev | CTTGCCACCGGTCTCCTTGCGGGCGGT |
| 2279 | H3-F forward | GGACTAGTGGATGGCCCGCACTAAGCAG |
| 2280 | H3-F reverse | CCTTAATTAAGTTGCGCTCACCGCGGAG |
| 2281 | H3-G forward | GAAGATCTATGGCCCGCACTAAGCAG |
| 2282 | H3-G reverse | GCTCTAGAGTTGCGCTCACCGCGGAG |
