# Supplementary material for: Substitutions in the Amino-Terminal Tail of Neurospora Histone H3 Have Varied Effects on DNA Methylation
Source: PLoS Genet. 2011 Dec 29;7(12):e1002423. doi: 10.1371/journal.pgen.1002423 (PMC3248561; doi:10.1371/journal.pgen.1002423)
Supplement: Table S4 — Summary of DNA methylation analyses for strains containing wild-type and mutant histone H3. (DOCX) [file pgen.1002423.s010.docx]

**Table S4. Summary of DNA methylation analyses for strains containing wildtype and mutant histone H3^1^**

| **Residue** | **Substitution/Insertion** | **Random integration approach** | | | **Targetted integration at *his-3* locus** | | |
| --- | --- | --- | --- | --- | --- | --- | --- |
|  |  | **Methylated Regions** | | | **Methylated Regions** | | |
|  |  | **Ψ-63** | **2:B3** | **8:G3** | **2:B3** | **2:G12** | **8:A6** |
| Wildtype | - | 100 | 100 | 100 | 100 | 100 | 100 |
| *dim-2* | - | 0 | 0 | 0 | 0 | 0 | 0 |
| R2 | R2L | 100 | 90 | 100 | 100 | 100 | 100 |
| K4 | K4L | 100 | 100 | 100 | n.d. | n.d. | n.d. |
| A7 | A7M | n.d. | n.d. | n.d. | 100 | 100 | 100 |
| R8 | R8A | 90 | 60 | 63 | 100 | 90 | 100 |
| K9 | K9L | 50 | 90 | 40 | 90 | 100 | 93 |
| S10 | S10A | 90 | 75 | 65 | 90 | 90 | 90 |
|  | S10E | n.d. | n.d. | n.d. | 90 | 90 | 95 |
|  | S10G | n.d. | n.d. | n.d. | 100 | 100 | 100 |
| T11 | T11A | 25 | 75 | 40 | n.d. | n.d. | n.d. |
| G12 | G12P | 25 | 75 | 65 | 100 | 100 | 100 |
| G13 | +G13 | 25 | 75 | 40 | n.d. | n.d. | n.d. |
| G13 | G13M | 25 | 90 | 40 | 90 | 100 | 100 |
| K14 | K14Q | 90 | 90 | 63 | 90 | 90 | 95 |
| A15 | A15M | n.d. | n.d. | n.d. | 100 | 100 | 100 |
| P16 | P16A | n.d. | n.d. | n.d. | 90 | 100 | 100 |
| R17 | R17L | n.d. | n.d. | n.d. | 90 | 100 | 100 |
| K18 | K18R | 100 | 100 | 100 | 100 | 100 | 100 |
| K23 | K23R | n.d. | n.d. | n.d. | 100 | 100 | 100 |
| K27 | K27L | n.d. | n.d. | n.d. | 100 | 100 | 100 |
| S28 | S28A | n.d. | n.d. | n.d. | 100 | 100 | 100 |
| K36 | K36L | n.d. | n.d. | n.d. | 100 | 100 | 100 |

**^1^** Data presented in Figure 1C, 3C, S2A, S2B and S4

**^2^** Number indicates % loss

n.d. Not Determined
