# Supplementary material for: Substitutions in the Amino-Terminal Tail of Neurospora Histone H3 Have Varied Effects on DNA Methylation
Source: PLoS Genet. 2011 Dec 29;7(12):e1002423. doi: 10.1371/journal.pgen.1002423 (PMC3248561; doi:10.1371/journal.pgen.1002423)
Supplement: Text S1 — Supplementary Experimental Methods. (DOCX) [file pgen.1002423.s011.docx]

**Text S1**

***In vitro* mutagenesis of the Neurospora histone H3 gene (*hH3*)**

Amino acid substitutions in the N-terminal tail of histone H3 were performed by the PCR-based QuickChange^TM^ Site-Directed Mutagenesis protocol (Stratagene) with pSH12 as template; this plasmid includes the wild type *hH3* gene with 1070 bp of the upstream region [[1](#_ENREF_1)]. Substitutions include R2L, K4L, T6A, A7M, R8A, S10A, T11A, G12P, G13M, K14R, K14Q, A15M, P16A, R17L, K18R, K18Q, K23R, K23Q, K27L, S28A, K36L. Allele +G13 contains one extra glycine codon (GGC) inserted between codons for residues 12 and 13. Primer pairs for mutagenesis are listed in Table S3. The K9L, K9R, S10G and S10E alleles were generated previously [[2](#_ENREF_2),[3](#_ENREF_3)]. All mutations were confirmed by DNA sequencing at the University of Oregon Genomics and Proteomics facility.

**Generation and purification of recombinant H3 variant proteins**

To generate recombinant GST-tagged histone H3 for *in vitro* methyltransferase assays, a DNA fragment corresponding to amino acid residues 1-57 of the histone H3 gene was amplified from pSH12 with primers H3(1-57)EX-FWD and H3(1-57)EX-REV. The resulting PCR product was digested with *Eco*RI and *Bam*HI, gel-purified and cloned into the GST expression vector pGEX-2T (Pharmacia) in *E. coli* strain BL-21 (DE3) Codon plus RIL (Stratagene). Amino acid replacements (K4L, A7M, R8A, K9L, S10A, T11A, G12P, G13M, K14R, A15M, P16A) were generated in this backbone with the QuickChange^TM^ Site-Directed Mutagenesis protocol (Stratagene) as described above. To prepare recombinant GST-tagged H3 proteins, 100 ml of LB medium with ampicillin (400 μg/ml) and chloramphenicol (34 μg/ml) was inoculated with *E. coli,* grown for 2.5 h hours at 37°C, shifted to room temperature for 30 min and expression of the GST-H3 fusion proteins was induced with 0.4 mM IPTG for 16 h. The cells were collected by centrifugation, lysed in 1 ml ice-cold RIPA buffer with 5 mg/ml lysozyme (Sigma) and subjected to sonication. Cell lysates were clarified by centrifugation and incubated for 1 hour with 500 μl glutathione agarose (Sigma). The agarose beads were washed four times with 25 ml RIPA buffer and the GST-H3 fusion proteins were eluted using 2 ml of 20 mM reduced glutathione (Sigma) prepared in 100 mM Tris-HCl (pH 8.5). Proteins were concentrated using Centricon-30 filter (Millipore) and concentration was measured by Bradford assays (Bio-Rad).

**Generation of Neurospora strains carrying mutated *hH3* genes**

Mutated *hH3* genes were introduced at ectopic location in the genome by two different approaches. In the first approach we used a Neurospora strain (N644) harboring a methylated and silent allele of a reporter gene that encodes for resistance to hygromycin (*hph*) [[4](#_ENREF_4)]. Strain N644 was co-transformed with mutant *hH3* genes and a dominant selectable marker (*Ben^R^*) that encodes for resistant to benomyl as described previously [[5](#_ENREF_5)]. Briefly, plasmids containing the mutant genes or the wild-type control (pSH12) were linearized with *Xba*I and co-transformed into strain N644 along with *Hin*dIII linearized plasmid pBT6 (*Ben^R^*). The transformants were grown *en masse* on solidified Vogel’s sucrose medium containing benomyl (0.5 μg/ml) and the resulting conidia were plated on media containing no drug, benomyl (0.5 μg/ml) or hygromycin (200 μg/ml) to test for reactivation of the *hph* gene. Random transformants were isolated from media containing hygromycin or benomyl and grown in liquid medium lacking drugs to isolate DNA. The presence of ectopic *hH3* copies was verified by Southern hybridization.

The second approach involved targeting mutant *hH3* genes to a known position in the genome, the *his-3* locus. For this purpose we used a strain of Neurospora that has a null allele of *hH3* (*hH3^RIP1^*) generated by RIP at the endogenous locus, and an allele in which serine 10 was replaced with alanine (*hH3^S10A^*) at the *his-3* locus [[3](#_ENREF_3)]. This strain (N3481) also has the gene coding for GFP-tagged HP1 at the *pan-2* locus. To generate *his-3* targeting vectors with desired substitutions in *hH3* (*hH3^X^*), we used a plasmid (pSH14) that contains wild type *hH3* gene in a *his-3* targeting vector [[1](#_ENREF_1)]. Plasmids bearing desired mutations that were generated by site-directed mutagenesis (described above) were digested with *Sph*I + *Mlu*I to recover fragments bearing these mutations and then sub-cloned into pSH14 digested with the same enzymes. The resulting plasmids were sequenced to confirm the presence of mutations, linearized by digestion with *Nde*I/*Dra*I and targeted to *his-3* region of strain N623 by electorporation [[6](#_ENREF_6)]. His^+^ transformants were screened by Southern hybridizations and crossed to strain N3481. His^+^ progeny from these crosses were screened by PCR-RFLP and Southern hybridzations to obtain suitable progeny that had histone H3 gene with desired mutation (*hH3^X^*) at the ectopic *his-3* locus and wild type (*hH3^WT^*) or null allele (*hH3^RIP1^*) at the endogenous locus. The ectopic *hH3^X^* alleles at *his-3* locus were amplified with primers that recognize the *his-3* flanks (pSH14-1 and pSH14-2) and the endogenous allele at the native locus was amplified with primers that recognize regions outside the duplication (H3-E Forward and H3-E Reverse). These PCR products were sequenced with primers listed in Table S3. The selected strains also harbored the gene coding for HP1-GFP at the *pan-2* locus. In order to confirm that the endogenous *hpo^+^* gene was not mutated by RIP, it was amplified with primers that recognize the flanks of the endogenous gene and do not overlap with the duplication at *pan-2*. The same primers were used to sequence these PCR products and are listed in the Table S3.

## Chromatin Immunoprecipitation

Chromatin immunoprecipitation (ChIP) experiments were performed as described previously [[7](#_ENREF_7)]. Antibodies used were: α-H3K9me3 (Active Motif LP Bio #AR-0170-10) and α-GFP (Abcam ab 290).

**Text S1 References**

1. Hays SM, Swanson J, Selker EU (2002) Identification and characterization of the genes encoding the core histones and histone variants of *Neurospora crassa*. Genetics 160: 961-973.

2. Tamaru H, Zhang X, McMillen D, Singh PB, Nakayama J, et al. (2003) Trimethylated lysine 9 of histone H3 is a mark for DNA methylation in Neurospora crassa. Nat Genet 34: 75-79.

3. Adhvaryu KK, Selker EU (2008) Protein phosphatase PP1 is required for normal DNA methylation in Neurospora. Genes Dev 22: 3391-3396.

4. Irelan JT, Selker EU (1997) Cytosine methylation associated with repeat-induced point mutation causes epigenetic gene silencing in *Neurospora crassa*. Genetics 146: 509-523.

5. Tamaru H, Selker EU (2001) A histone H3 methyltransferase controls DNA methylation in Neurospora crassa. Nature 414: 277-283.

6. Margolin BS, Freitag M, Selker EU (1997) Improved plasmids for gene targeting at the *his-3* locus of *Neurospora crassa* by electroporation. Fungal Genetics Newsl 44: 34-36.

7. Honda S, Selker EU (2008) Direct interaction between DNA methyltransferase DIM-2 and HP1 is required for DNA methylation in Neurospora. Mol Cell Biol 28: 6044–6055.
